# Unraveling the Omega-3 Puzzle: Navigating Challenges and Innovations for Bone Health and Healthy Aging

**DOI:** 10.3390/md22100446

**Published:** 2024-09-28

**Authors:** Zayana Ali, Mohammad Ahmed Al-Ghouti, Haissam Abou-Saleh, Md Mizanur Rahman

**Affiliations:** 1 Biological Science Program, Department of Biological and Environmental Sciences, College of Arts and Sciences, Qatar University, Doha P.O. Box 2713, Qatar; za2211104@qu.edu.qa; 2 Environmental Science Program, Department of Biological and Environmental Sciences, College of Arts and Sciences, Qatar University, Doha P.O. Box 2713, Qatar; mohammad.alghouti@qu.edu.qa; 3 Biomedical Sciences Department, College of Health Sciences, Qatar University, Doha P.O. Box 2713, Qatar; hasaleh@qu.edu.qa

**Keywords:** omega-3 polyunsaturated fatty acids, eicosapentaenoic acid (EPA), docosahexaenoic acid (DHA), bone health, aging, bioavailability, nanoparticle encapsulation, specialized proresolving mediators (SPM)

## Abstract

Omega-3 polyunsaturated fatty acids (ω-3 PUFAs, n-3 PUFAs), including eicosapentaenoic acid (EPA), docosahexaenoic acid (DHA), and alpha-linolenic acid (ALA), are essential polyunsaturated fats primarily obtained from fatty fish and plant-based sources. Compelling evidence from preclinical and epidemiological studies consistently suggests beneficial effects of ω-3 PUFAs on bone health and healthy aging processes. However, clinical trials have yielded mixed results, with some failing to replicate these benefits seen in preclinical models. This contraindication is mainly due to challenges such as low bioavailability, potential adverse effects with higher doses, and susceptibility to oxidation of ω-3 fatty acids, hindering their clinical effectiveness. This review comprehensively discusses recent findings from a clinical perspective, along with preclinical and epidemiological studies, emphasizing the role of ω-3 PUFAs in promoting bone health and supporting healthy aging. Additionally, it explores strategies to improve ω-3 PUFA efficacy, including nanoparticle encapsulation and incorporation of specialized pro-resolving mediators (SPM) derived from DHA and EPA, to mitigate oxidation and enhance solubility, thereby improving therapeutic potential. By consolidating evidence from various studies, this review underscores current insights and future directions in leveraging ω-3 PUFAs for therapeutic applications.

## 1. Introduction

Nutritional factors play a crucial role in determining health outcomes. An inadequate diet is associated with various health problems such as decreased bone mineral density, cardiovascular disease, and neurodegenerative disorders [1]. A targeted nutritional approach that includes key bioactive substances can significantly improve health outcomes. Recent studies have unveiled novel insights into the supplementation of omega-3 fatty acids, highlighting their increasing recognition for health benefits.

**Figure 1:**
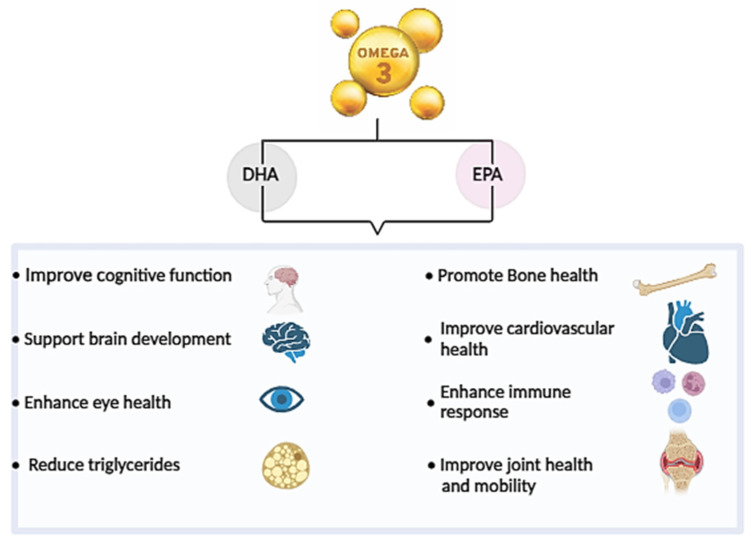
Clinical benefits of Omega-3 Fatty Acids DHA and EPA.

Omega-3 fatty acids are essential polyunsaturated fatty acids with a molecular structure containing multiple double bonds. They are classified based on the position of the first double bond from the methyl end (omega, ω- or n- end) of the fatty acid chain. In the case of ω-3 PUFAs, this first double bond is located between the third and fourth carbon atoms from the tail end [2]. ω-3 PUFAs, including docosahexaenoic acid (DHA C22:6 ω-3), eicosapentaenoic acid (EPA, C20:5 ω-3), docosapentaenoic Acid (DPA C22:6 ω-3), and α-Linolenic acid (ALA, C18:3 ω) offer a wide range of biological effects on human health. These effects include managing neurological disorders, cardiovascular diseases, enhancing cognitive abilities, regulating skeletal muscle mass, maintaining membrane permeability, supporting neurogenesis, aiding in eye development, reducing bone mineral density loss, combating various cancers, improving muscle function and alleviating inflammatory conditions [3,4,5,6,7,8,9,10,11] (Figure 1). A study showed that supplementation with fish oil high in EPA/DHA content (oil C) is more effective in reducing inflammation compared to regular fish oils and corn oil in C57BL/6 female mice, as it significantly decreases levels of pro-inflammatory cytokines in macrophages [12].

Humans cannot synthesize ω-3 PUFAs internally due to the lack of specific enzymes required for introducing the ω-3 double bond at the methyl end of fatty acids, opposite the carboxylic acid group [13]. Therefore, ω-3 PUFAs are vital nutrients that need to be acquired through dietary sources. They are abundantly present in marine sources such as fish (salmon, tuna, halibut), marine organisms (algae, krill), and certain plant-derived oils (flaxseed, canola oil, soybeans). The main source of EPA and DHA is cold-water fish, which usually have greater body fat percentages [14]. ALA is derived from plant-based sources such as flaxseed, canola (rapeseed) oil, soybeans, pumpkin seeds, perilla seed oil, walnuts, and their oils [15].

ALA can be converted in the body to EPA and DHA through elongation and desaturation reactions; this conversion efficiency is limited. Research indicates that only a small percentage of ALA is converted into EPA and even less into DHA, especially in males. [16]. In males, the conversion rate of ALA to EPA and DHA is reported to be less than 8% and 4%, respectively, while in females, these rates are slightly higher at approximately 21% and 9%, respectively [17]. Consuming whole foods rich in omega-3s, such as fatty fish, is the best way to ensure adequate intake. However, for those with limited fish consumption, it is essential to consume preformed EPA and DHA to significantly increase their levels in biological tissues [18]. Recommended methods include using pharmaceutical-grade supplements containing synthetic EPA and DHA, either separately or in combination [19].

Studies have shown that ω-3 PUFAs positively impact bone health by promoting bone formation, enhancing peak bone mass, and attenuating bone density loss across diverse populations, including postmenopausal women and individuals with osteoporosis [20,21,22,23]. Elevated plasma marine ω-3 PUFAs levels are associated with higher bone mineral density (BMD) and a lower fracture risk [24,25]. A significant correlation was seen between total bone mineral density and elevated serum phospholipid DHA levels in healthy males aged 16 to 22 [26]. Additionally, ω-3 PUFAs have shown promise in managing and preventing osteoporosis, a condition characterized by bone loss and increased fracture risk, which remains a significant global health concern [27,28]. Approximately 4.3 million new osteoporotic fractures occurred in Europe in 2019, equating to eight new fractures every minute. The prevalence of osteoporosis is 29.0% in Chinese women, 13.5% in Chinese men over 50, 16% in Caucasian women over 50, and 39.3% in Spanish elderly in 2020 [27,29,30]. Lower incidences of osteoporosis are seen in populations that consume large amounts of seafood high in ω-3 PUFAs, such as the Japanese and Greenland Eskimos [31].

Additionally, growing evidence from studies suggests that ω-3 PUFAs offer significant neuroprotective effects against age-related neurodegenerative diseases, including Alzheimer’s disease (AD) and various forms of dementia [32,33]. AD, a progressive neurodegenerative disorder affecting millions globally, is estimated to have impacted approximately 6.5 million Americans aged 65 and older in 2021 [34,35]. While current medications provide some relief for mild-to-moderate symptoms, they lack the ability to slow disease progression. Mounting evidence suggests a potential therapeutic role for ω-3 PUFAs in age-related AD [36]. Deficiency in ω-3 PUFAs has been linked to an increased risk of AD and depression in older adults (>75 years old) [37], while supplementation with EPA and DHA has shown potential in improving cognitive function and reducing AD risk in healthy women over 60 [38].

Despite their advantages, food fortification with ω-3 PUFAs presents several challenges due to their low water solubility, rapid oxidation, potential adverse effects at high doses, and variable bioavailability [39,40]. These issues can lead to reduced shelf life, diminished consumer acceptability, compromised functionality, and decreased nutritional value and safety. To address these challenges, several solutions can be employed. Advanced encapsulation technologies are one approach, involving the incorporation of omega-3 oils into well-designed colloidal particles made from food-grade ingredients, such as liposomes, emulsion droplets, nanostructured lipid carriers, or micro gels [41]. Another effective solution is using specialized proresolving mediators derived from ω-3 PUFAs, which are more bioavailable and exhibit therapeutic effects even at very low doses [42].

In this article, we begin by reviewing the possible health benefits of ω-3 PUFAs in bone health and healthy aging, covering results from various preclinical, clinical, and epidemiological studies. We then highlight the main challenges associated with incorporating these fatty acids. Finally, we discuss the use of nanoparticle encapsulation and the potential of specialized proresolving mediators to enhance the performance of ω-3 PUFAs.

## 2. Omega-3 Fatty Acids and Bone Health

Bone remodeling is a dynamic process involving bone formation and resorption. Pre osteoblasts, derived from bone marrow stem cells, mature into osteoblasts, which are essential for bone formation. Osteoclasts, specialized for bone resorption, break down bone tissue to regulate calcium levels and shape bone structure [43]. When bone resorption surpasses bone formation, it results in bone loss and microstructural damage, potentially leading to osteoporosis [44].

**Figure 2:**
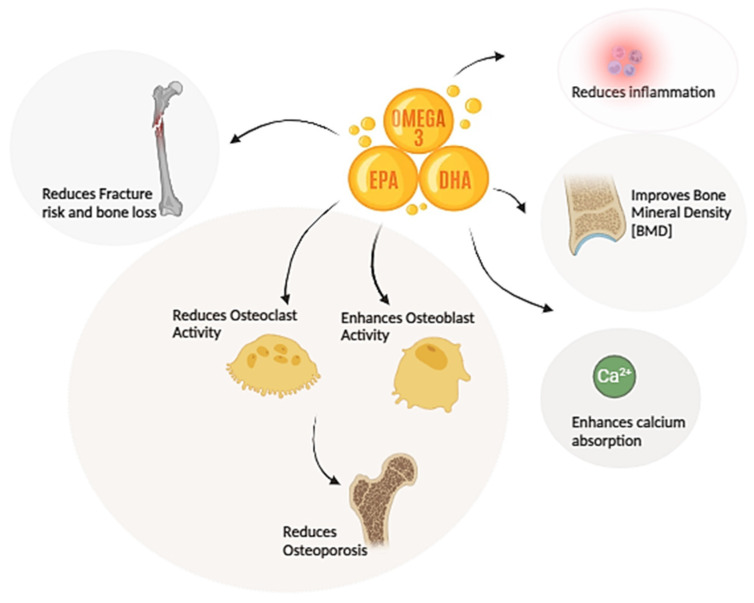
Impact of ω-3 PUFAs on Bone Health.

Dietary fats significantly influence bone health through various mechanisms, such as altering calcium absorption, prostaglandin synthesis, and regulating osteoclastogenesis (bone resorption) and osteoblastogenesis (bone formation) [45] (Figure 2). Among these fats, ω-3 PUFAs play a particularly crucial role due to their ability to regulate PPARγ-mediated adipocyte differentiation [46] modulate inflammatory responses [47] and enhance bone marrow microcirculation [48]. These processes are crucial for maintaining skeletal integrity and overall bone health, highlighting the importance of dietary fat composition in bone metabolism. Moreover, ω-3 PUFAs have been demonstrated to reduce fracture risk [49], prevent bone loss [50], and improve bone mineral density (BMD) [51,52], further emphasizing their significance in bone health.

Inflammatory cytokines are important in promoting osteoclastic bone resorption [53]. Given their established anti-inflammatory properties and positive influence on bone health, ω-3 PUFAs could potentially exert a protective effect by suppressing Osteoclastogenesis [54]. High ω-3 PUFAs consumption may increase bone formation by modifying the synthesis of bone growth factors and directly alter the lipid profile in bone marrow by increasing ω-3 PUFAs concentrations in bone marrow [55]. Elevated prostaglandin E2 (PGE2) levels have been linked to decreased osteoprotegerin (OPG) production and increased receptor activator of nuclear factor kappa-Β ligand (RANKL) expression, both of which can negatively impact bone health by promoting osteoclast formation [56]. Interestingly, ω-3 PUFAs may counteract this process by potentially promoting intestinal calcium absorption while simultaneously inhibiting PGE2 synthesis [57]. Their ability to promote bone formation and inhibit bone resorption makes them crucial for maintaining bone health and preventing osteoporosis [58].

### 2.1. Preclinical Studies

Supplementing with ω-3 PUFAs has been demonstrated to improve bone mass, BMD, and bone strength in animal models [59,60]. In vitro studies highlights that EPA and DHA influence the maturation, activity, and survival of bone cells, promoting bone formation and inhibiting the bone resorption processes [61,62,63]. Animal studies, particularly in models of ovariectomy, suggest that dietary supplementation with fish oil, rich in ω-3 PUFAs, can prevent bone loss by enhancing mineral apposition and maintaining bone density [64,65]. Additionally, rodent studies demonstrate that diets enriched with DHA improve femur BMD, whereas diets with high n-6 ratios show less favorable effects on bone health [66,67]. Moreover, diets high in ω-3 PUFAs have been shown to mitigate bone loss, osteoclast formation, and bone marrow adiposity in aged animals with osteoporosis [68,69]. Further investigations into maternal diets enriched with ω-3 PUFAs by Tompkins YH et al. revealed positive impacts on skeletal integrity and fat mass regulation in broiler offspring compared to those fed soybean oil [70].

A study showed that ω-3 PUFAs, by lowering the n-6/n-3 fatty acid ratio, significantly reduced pro-inflammatory responses in animal models [71]. Additionally, the study found that when ω-3 PUFAs were combined with calorie restriction, their anti-inflammatory effects were further enhanced, leading to a marked reduction in Nuclear factor-kappa B (NF-κB) activity. NF-κB is a vital multi-subunit transcription factor that controls the expression of pro-inflammatory cytokines and enzymes, which are crucial in the development of chronic inflammatory diseases [72]. This combination also results in decreased secretion of pro-inflammatory cytokines such as IL-6 and TNF-alpha. Recent studies have highlighted the beneficial effects of krill oil (KO), another rich source of EPA and DHA [73], in reducing inflammation and improving cartilage health in models of Monosodium Iodoacetate-Induced osteoarthritis(OA) and rheumatoid arthritis [74]. Oral administration of KO has also been found to enhance cartilage strength and bone mineral density through promoting chondrocyte autophagy and reducing apoptosis in a surgical mouse model of knee OA [75]. According to research by Ku SK et al., administering KO orally for 8 weeks at doses of 200 and 100 mg/kg enhanced cartilage compressive strength and bone mineral density while also improving joint swelling and mobility in an animal model [76].

Studies focusing on the dietary balance of n-3 and ω-6 PUFAs underscore their impact on preventing osteoporotic osteoarthritis, emphasizing the importance of maintaining a low n-6/n-3 ratio for preserving cartilage structure and function. The effects of dietary PUFAs on osteoporotic osteoarthritis in dual-model mice were investigated by Dai Y et al. The researchers found that low n-6/n-3 PUFA ratios greatly enhanced cartilage structure and prevented the loss of polysaccharides found in articular cartilage [77]. Huang et al. demonstrated that increasing the n-3/n-6 PUFA ratio in articular cartilage of Fat-1 mice, which express an enzyme converting n-6 fatty acids into n-3 fatty acids [78,79], prevented the onset of osteoarthritis by significantly enhancing autophagy and promoting chondrocyte survival [80]. According to another study, a 20:1 n-6/n-3 PUFA diet with FO significantly reduced systemic inflammation and cartilage destruction in osteoporotic OA rats compared with the same diet without fish oil (FO) [81]. In a study, it was found that Fat-1 mice, with elevated n-3 fatty acids and a reduced n-6/n-3 fatty acid ratio in bone marrow phospholipids, were able to sustain higher bone mineral density (BMD) in estrogen-deficient conditions compared to wild-type (WT) mice [82].

Additionally, research by Zhu, Y et al. on the anti-osteoporotic effects of lipids derived from *Tilapia nilotica* fish head lipids (THLs), which are rich in EPA and DHA, in ovariectomized osteoporotic rats, demonstrated their protective effects against bone loss, increased bone mineral density, decreased bone resorption, and enhancement of bone microstructure in osteoporotic conditions [83].

An in vitro study by Cifuentes-Mendiola, S.E. et al. explored the impact of DHA on osteoblast mineralization under high glucose conditions using human osteoblast cell lines treated with 24 mM glucose (HG), alone or in combination with 10 or 20 µM DHA [84]. Their findings revealed that DHA effectively mitigated the adverse effects of high glucose on bone mineral matrix quality and reduced oxidative stress. These results suggest a protective role of DHA, even at relatively low doses, in maintaining bone health under elevated glucose levels, with potential therapeutic benefits for diabetic patients or those with impaired glucose metabolism. Additionally, recent studies by Cugno et al. demonstrated that ω-3 PUFAs supplementation restored the osteoblastic differentiation capacity of C3H10T1/2 cells and inhibited the up-regulation of osteoclast differentiation in RAW264.7 cells [85]. Various preclinical studies have demonstrated the beneficial impact of ω-3 FAs in promoting bone health, as summarized in Table 1.

### 2.2. Clinical Studies

Clinical studies exploring the effects of ω-3 PUFAs on bone health have yielded conflicting results. While some studies support the potential benefits of ω-3 PUFAs in enhancing bone metabolism and reducing the risk of osteoporosis, other studies report no significant effects. This section reviews the clinical evidence, highlighting both the positive outcomes and the limitations.

#### 2.2.1. Conflicting Evidence

Many clinical investigations have yielded conflicting results, suggesting that ω-3 PUFAs have no significant effect on bone health. For instance, Melanie et al. studied the effects of 1g/d of EPA and DHA, vitamin D3, and a home-based exercise program on BMD in healthy older adults over 3 years. They found no significant benefits of ω-3 PUFAs or home-based exercise on lumbar spine, femoral neck, or total hip BMD [91]. Similarly, Jorgensen et al. found no significant differences in bone mineral density (BMD) at various skeletal sites after 44 weeks of supplementation with 2.6 g of marine ω-3 PUFAs in kidney transplant recipients [92]. A meta-analysis revealed that ω-3 PUFAs supplementation slightly improves lumbar spine BMD by 2.6% but has no effect on femoral neck BMD [93]. Another study on pre- or post-menopausal women found no notable effect on BMD after 12 months of supplementation with 0.44g/d of marine fish oil [94]. LeBoff MS et al. found that participants taking 1 g/day of omega-3 supplementation with vitamin D for 2 years did not experience significant prevention of bone loss at the hip, spine, or whole body compared to those taking a placebo [95]. In a study by Rajaram S et al., neither changing the n-6 PUFA ratio from 10:1 to 2:1 nor supplementing with 1 g/day of the active form of EPA + DHA (1.40/5.04 g EPA/DHA) significantly affected bone turnover markers or PPAR-γ gene expression in healthy adults over 8 weeks [96]. Similarly, Chen et al. found no significant differences in 2-year BMD measures among participants with knee osteoarthritis receiving either high (4.5 g/day) or low (0.45 g/day) doses of omega-3 fish oil. Although concerns about increased bleeding events at higher doses of ω-3 PUFAs exist, their study supports the safety of fish oil at doses up to 4.5 g daily of EPA+DHA [97]. Another meta-analysis found no serious adverse effects with doses up to 1.86 g daily in elderly subjects [98]. Additionally, Appleton, K. M. et al. found no evidence linking ω-3 PUFAs supplementation to changes in bone resorption in mildly depressed individuals [99]. A study on Crohn’s disease patients found that high-dose fish oil (1.6 g EPA + 1.1 g DHA/day) and antioxidants for 24 weeks did not alter bone turnover or bone resorption [100]. Razny et al. also found no significant benefit on bone remodeling from 1.8g/day ω-3 PUFAs supplementation during weight loss [101].

#### 2.2.2. Positive Results

Despite the overall inconsistent findings, certain studies highlight the potential benefits of ω-3 PUFAs for bone health. For example, a preliminary pilot study involving 26 patients with OA reported that administration of EPA (10 mL) for 6 months resulted in a 30% reduction in interference with daily activities and a 37% reduction in pain ratings. Although these results lacked statistical significance due to the limited sample size, they suggest the potential efficacy of EPA in alleviating OA symptoms [102]. In another study, elderly women who received 6 g of mixed oils containing 0.42 g of marine ω-3 PUFAs for 18 months demonstrated increased hip BMD [103]. Similarly, Baggio et al. found a positive correlation between plasma ω-3 PUFAs levels and BMD in kidney transplant recipients over 2 years [93].

Some studies suggest potential benefits of ω-3 PUFAs under specific conditions. For instance, Hutchins-Wiese HL et al. found that high-dose fish oil supplementation (4 g of EPA and DHA) daily for 3 months can decrease bone turnover and reduce bone resorption in postmenopausal breast cancer survivors undergoing aromatase inhibitor therapy [104]. Similarly, other research indicated that DHA reduced breast cancer bone metastasis and associated osteolysis more effectively than EPA by inhibiting cancer cell migration to bone and suppressing osteoclastic bone resorption [105]. In a study on very low-birth-weight -VLBW neonates, researchers found that ω-3 PUFAs –enriched parenteral lipid emulsions resulted in a lower rate of OPG/sRANKL reduction, suggesting these emulsions may help mitigate early bone loss in VLBW neonates [106]. In a 12-month randomized, double-blind, placebo-controlled trial, Ichinose T et al. found that healthy elderly Japanese individuals consuming a DHA-enriched milk beverage showed a significant decrease in serum levels of TRACP-5b, a bone resorption marker, but also reduced serum levels of BAP, a marker for bone formation. This indicates that DHA may have complex and opposing effects on bone metabolism [107]. Similarly, Fonolla-Joya J et al. found that healthy postmenopausal women who consumed a dairy drink enriched with 40 mg/100 mL of EPA and DHA showed a positive impact on bone metabolism by decreasing serum levels of PTH and RANKL, indicating a potential reduction in bone resorption [108]. In another study involving hyperlipidemia adults, replacing regular milk with fortified milk containing 5.17 g oleic acid, 0.14 g DHA, and 0.20 g EPA per 500 mL from fish oils over 1 year significantly increased plasma EPA (by 42%) and DHA (by 60%). This dietary change also enhanced bone-formation markers like OPG and osteocalcin, suggesting potential benefits for bone health [109].

Studies examining the relationship between n-3 FAs and hip fracture risk highlight promising avenues in bone health research. In a case-control and cross-sectional study involving Korean hip surgery patients, higher plasma levels of n-3 FAs correlated with reduced odds of osteoporotic hip fracture (HF) and enhanced bone mass. Notably, these FAs were found to suppress Osteoclastogenesis, evident from decreased TRAP-5b levels in bone marrow aspirates. This suggests a potential role for n-3 FAs in regulating bone health by mitigating osteoclast differentiation and bone resorption [110]. Similarly, Orchard TS et al. conducted a case-control study focusing on postmenopausal women, revealing that elevated levels of RBC ALA, EPA, and total ω-3 PUFAs correlated with a lower risk of hip fracture. Conversely, a high ratio of n-6 to n-3 FAs in RBCs was associated with an increased risk of hip fracture [111]. These case-control studies contribute valuable insights into the protective effects of n-3 FAs against hip fracture risk, highlighting their role in bone health maintenance and fracture prevention. These findings are summarized in Table 2.

The divergent outcomes underscore the complexity of ω-3 PUFAs role in bone metabolism and the importance of continued investigation to better understand their potential therapeutic benefits.

### 2.3. Epidemiological Studies

Epidemiological studies indicate that regular consumption of ω-3 PUFAs is positively correlated with better bone health in older adults. These studies have assessed the impact of PUFAs, primarily through food frequency questionnaires (FFQs), highlighting a protective effect of higher intake of ω-3 PUFAs (EPA/DHA and ALA) and a lower n-6 PUFA ratio on bone health [118].

In contrast, studies comparing different populations reveal variations in the impact of fish and shellfish consumption on bone health. Choi and Park et al. found that higher fish and shellfish intake had protective effects against bone loss, whereas similar associations were less pronounced among American populations [119]. The studies shows significant discrepancies in fish and shellfish consumption between Asian [120,121] and American populations [122]. Effective intake for maintaining BMD in Asians ranged from 250 to 833 g/week, with elderly Koreans averaging around 353 g/week [123]. Conversely, Americans exhibited notably lower average intakes, ranging from approximately 113.5 to 158 g/week [124]. Higher intake among Koreans suggests a protective effect against bone loss. These variations highlight potential disparities in bone health outcomes between the two groups, emphasizing the significance of dietary patterns in osteoporosis prevention. In a previous epidemiological study, Japanese populations with an average intake of 2.7 g/day of ω-3 PUFAs showed a positive association between ω-3 PUFAs intake and BMD [51]. Studies underscore a positive association between higher marine fish intake and enhanced BMD, particularly among Chinese women consuming substantial quantities. In a study by Chen YM et al., Chinese women in the highest quintile for marine fish intake (65 g/day) had significantly higher BMD compared to those in the lowest quintile (0.6 g/day) and the combined quintiles 2–4 (16.8 g/day) [125]. In another study, Chinese individuals consuming over 502 g/week of fish and shellfish demonstrated lower risks of hip fractures [126]. Moreover, Amerindian women who consume ≥ 5.2 servings (728 g) of fatty fish per week report lower incidences of age-related osteopenia and osteoporosis [127]. In contrast, Europeans with an average intake of 140 g/week showed no significant association between fish intake and hip fracture risk [128]. In Spanish women aged 20–79, a study by Lavado-García et al. found that DHA intake was notably associated with increased lumbar spine BMD in individuals without bone density issues. However, no significant associations were observed between long-chain ω-3 PUFAs and lumbar spine BMD in women who were osteopenic or osteoporotic [129]. These findings collectively highlight the complex but generally positive role of ω-3 PUFAs in maintaining and improving bone health across different populations.

Intake of ω-3 PUFAs has been linked to various markers of bone health. Studies show that higher levels of ω-3 PUFAs correlate positively with serum N-terminal propeptide of type I collagen, a marker indicating bone production. Conversely, these fatty acids are inversely related to urinary type-1 collagen cross-linked-N-telopeptide (NTx), which is a marker of bone resorption) [51]. Additionally, ω-3 PUFAs have been inversely associated with OA in adults aged 40 to 59, and sufficient intake of total ω-3 PUFAs is suggested to optimize peak bone mass (PBM) at the hip in females aged 25 years and older [130,131]. Favorable low n-6:n-3 ratio, along with higher intake of long-chain ω-3 PUFAs, is associated with higher bone mineral density (BMD) in the femur and reduced bone resorption [132]. Research further highlights positive correlations between BMD and the intake of various types of fatty acids, including saturated, monounsaturated, and polyunsaturated fatty acids. Notably, an intake exceeding 20.52 g/day of monounsaturated fatty acids (MUFAs) significantly correlates with improved BMD, underscoring the benefits of ω-3 PUFAs for bone health [4]. These findings are summarized in Table 3.

## 3. Omega-3 Fatty Acids and Aging

Aging is characterized by a series of progressive and irreversible biological changes that significantly influence health outcomes [136]. Moreover, aging is a recognized causative factor in the development of neurodegenerative diseases such as Alzheimer’s disease (AD) and contributes to cognitive decline [137]. Cognitive decline, in particular, emerges as a significant concern, with projections indicating that the prevalence of dementia, a condition characterized by substantial cognitive impairment [138], could rise to 115.4 million individuals by 2050 [139]. Over the past 2 decades, cognitive impairment resulting from AD has emerged as a significant factor, significantly impacting the quality of life for patients, and posing a substantial threat to healthcare resources [140].

**Figure 3:**
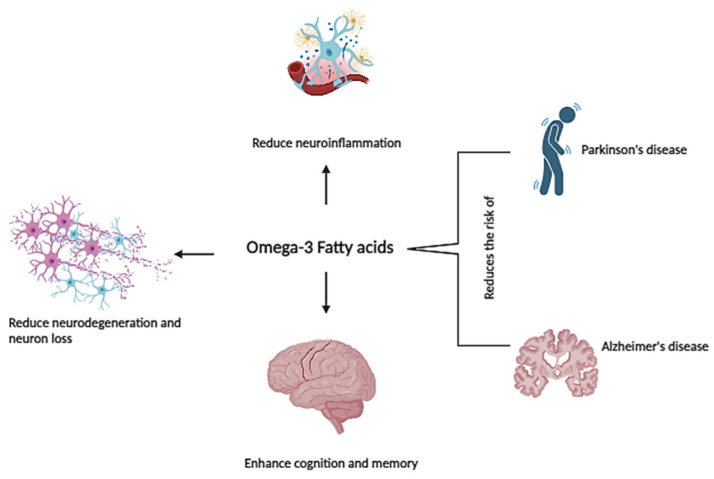
Impact of Omega-3 Fatty Acids on Age-Related Neurodegenerative Diseases.

ω-3 PUFAs, specifically the long-chain varieties DHA and EPA, are pivotal for brain health. Increased intake of these ω-3 LCPUFAs has been associated with improved cognitive function, decelerated cognitive decline, and a decreased risk of developing dementia [141] (Figure 3). Moreover, DHA and EPA show promise as bioactive ingredients in treating more severe age-related neurological diseases such as Alzheimer’s and Parkinson’s disease [142,143]. The decline of ω-3 PUFAs has been observed in the natural aging process, correlating with brain atrophy and memory impairment [144]. Several epidemiological studies generally suggest that higher consumption of DHA may potentially offer protective benefits against cognitive decline in older adults. However, the conclusions drawn from multiple review articles indicate that the association between ω-3 PUFAs intake and cognitive decline remains inconclusive [145,146]. Specifically, clinical studies involving healthy subjects have not consistently shown a direct link between supplementation with ω-3 LCPUFAs and improvements in cognitive function during the aging process [143].

### 3.1. Preclinical Studies

Several preclinical studies have illuminated the diverse roles of ω-3 PUFAs in mitigating age-related conditions, spanning from neuroprotection in Alzheimer’s disease to preserving visual function and liver health during aging.

Inflammation and oxidative stress are linked to aging and a wide range of diseases, including neurodegenerative disorders. Strategies targeting both inflammation and oxidative stress are essential for reducing pathological conditions and promoting healthy aging. A study found that Fat-1 mice, which possess the ability to convert n-6 fatty acids into n-3 fatty acids when combined with calorie restriction (CR), showed significantly higher levels of peroxisome proliferator-activated receptor (PPAR)gamma and sirtuin (SIRT)-1 in the liver compared to wild-type (WT) mice subjected to the same CR regimen [147]. These findings suggest that combining n-3 fatty acids with calorie restriction can effectively reduce inflammation and oxidative stress, potentially improving health and extending lifespan with aging. DHA has proven to be more effective than EPA in addressing aging-related inflammatory diseases, as shown in a study using a mouse model of systemic lupus erythematosus (SLE) [148]. The research demonstrated that fish oil enriched with DHA (FO-DHA) substantially reduced serum anti-dsDNA antibodies, kidney IgG deposition, and proteinuria, while also inhibiting inflammatory pathways including IL-18 induction and IL-18–dependent signaling. These results highlight DHA’s superior ability to mitigate inflammation and prolong lifespan compared to EPA-enriched fish oil. Additionally, aging can also impact pain sensitivity and response, with chronic pain often worsening quality of life and contributing to depression. A study investigated the long-term effects of concentrated n-3 fatty acid-rich fish oil (CFO), regular fish oil (FO), and n-6 fatty acid-rich safflower oil (SO) on thermal pain sensitivity in aging mice, finding that CFO significantly reduces pain sensitivity [149]. This highlights the importance of ω-3 PUFAs supplementation in managing pain associated with aging.

Wu K et al. investigated the therapeutic potential of ω-3 PUFAs in AD. They employed C57BL/6 transgenic mice, which carry the fat-1 gene to increase endogenous ω-3 PUFAs by converting n-6 PUFAs to ω-3 PUFAs, crossed with amyloid precursor protein (APP) transgenic mice, to evaluate the protective effects of endogenous ω-3 PUFAs on cognitive and behavioral deficits. The study suggests that maintaining enriched ω-3 PUFAs in the brain mitigate cognitive decline and prevent neuropsychological impairments in AD [150]. Expanding on the benefits of ω-3 PUFAs, Wang reported that 0.33% DHA milk supplementation reduced oxidative stress in SAMP8 mice, as evidenced by enhanced Superoxide Dismutase (SOD) antioxidant enzyme activity [151]. Although exogenous and endogenous DHA milk had identical DHA dosages, they exhibited similar effects on oxidative stress indicators but differed in cognitive function in SAMP8 mice. Endogenous DHA milk had superior effects on oxidative stress and cognition compared to exogenous DHA, enhancing mice’s cognitive ability with increased platform crossings in the water maze test. This disparity underscores potential differences in bioavailability, as the uptake and utilization of different DHA forms varies [151]. Additionally, neuroinflammation due to microglial and astrocyte polarizations can lead to neurodegeneration and cognitive decline. Increased microglial M1 and astrocytic A1 polarizations elevate brain pro-inflammatory cytokines. In their study, Xia J. et al. found that DHA and EPA enhanced spatial memory in aging rats, with DHA showing greater effectiveness [152]. DHA was also superior in reducing aging-related neuroimmunological changes, thus more significantly improving memory impairment. Beyond cognitive benefits, age-related declines in visual function are also a concern among disease-free individuals, with lipofuscin accumulation playing a pivotal role in age-related vision impairments [153]. Recent studies in aged mice indicate that omega-3 supplementation mitigates retinal lipofuscin accumulation and protects photoreceptors, suggesting potential benefits in slowing age-related retinal degeneration [154]. Studies into the aging process often emphasize the liver’s resilience, yet recent research has highlighted nuanced effects based on dietary fat sources. Varela-Lopez et al. explored the lifelong consumption of various fat sources in aged rats, including monounsaturated fatty acids (virgin oil), n6 polyunsaturated fatty acids (sunflower oil), and n3 polyunsaturated fatty acids (fish oil) [155]. Among these, virgin olive oil, rich in MUFAs, emerged as particularly beneficial for liver preservation during aging.

Building on this, the role of dietary fats in aging extends beyond liver health. Telomere shortening, which is closely linked to mortality and age-related diseases, can be mitigated by ω-3 PUFAs. Chen J. et al. demonstrated that ω-3 PUFAs protect the liver and testes from telomere shortening by 13–25% and 25–27%, respectively, while also effectively reducing D-galactose-induced oxidative stress by boosting antioxidant activities and lowering plasma F2-isoprostane levels [156].

Collectively, these preclinical findings (Table 4) underscore the potential of ω-3 PUFAs to mitigate age-related declines and promote healthy aging.

### 3.2. Clinical Studies

Clinical studies exploring the relationship between ω-3 PUFAs and aging have unveiled intriguing insights into their potential impact on cognitive health and neurodegenerative diseases. Most of the research spanning cognitive impairment, AD, and Parkinson’s disease (PD) suggests that ω-3 PUFAs, particularly DHA and EPA, play pivotal roles in mitigating cognitive decline, enhancing memory function, and potentially slowing disease progression.

In a study by Lin P.Y. et al., supplementation with ω-3 PUFAs in patients with cognitive impairment did not show significant improvements in general cognitive, functional, or mood status compared to placebo after 24 months. However, they observed enhancements in spoken language ability and constructional praxis sub items of the ADAS-cog, suggesting a potential selective cognitive benefit [33]. Sinn et al. investigated the effects of 6 months’ supplementation with EPA-rich fish oil and DHA-rich fish oil on older adults with mild cognitive impairment (MCI). They suggest that increased intake of DHA and EPA may benefit mental health, reduce depressive symptoms, and potentially lower the risk of progression to dementia in this population. However, no effects were observed on other cognitive or quality-of-life parameters [157]. Moreover, in another study, researchers found that supplementing healthy older adults experiencing mild memory complaints with 0.9g/d of DHA improved their episodic memory and learning abilities [158]. These findings suggest that DHA supplementation can enhance memory function in individuals affected by age-related cognitive decline (ARCD).

Recent research indicates that increasing dietary consumption of carotenoids and ω-3 PUFAs reduce the incidence of cognitive decline and dementia in older individuals [159]. Additionally, Arellanes et al. highlighted that APOE4 carriers may have reduced brain supply of DHA and EPA, suggesting that higher doses of DHA may be necessary for optimal brain bioavailability. They demonstrated that doses of TG-DHA at 2 g/day or more were effective, particularly in APOE4 carriers, whereas lower doses had limited brain delivery [160]. Torres-Mendoza et al. found that stable doses of DHA and EPA reduced plasma protein and lipid oxidation while increasing catalase activity, with no significant change in superoxide dismutase activity [161]. In a recent study, researchers highlighted the potential benefits of omega-3 and vitamin E co-supplementation in modulating specific inflammatory and metabolic pathways in PD patients [162]. They found that 12 weeks of ω-3 PUFA and vitamin E co-supplementation in elderly subjects with PD significantly improved the gene expression of TNF-α, PPAR-γ, and LDLR. However, there was no significant effect on the gene expression of IL-1 and IL-8. Additionally, Taghizadeh et al. reported significant improvements in UPDRS (Unified Parkinson’s Disease Rating Scale) scores in PD patients following ω-3 PUFA and vitamin E supplementation [163]. Furthermore, Pantzaris et al. investigated Neuroaspis PLP10™, a supplement rich in omega-3 and omega-6 fatty acids with antioxidant vitamins, in early Parkinson’s disease. They found that supplementing PD patients with Neuroaspis PLP10™ for 30 months slowed disease progression, based on UPDRS-I scores, suggesting potential neuroprotective effects [164]. Additionally, Del Brutto O.H. et al. observed an inverse relationship between oily fish intake and progression of white matter hyper intensities (WMH), a marker of cerebral small vessel disease (cSVD) and cognitive decline, in frequent fish consumers of Amerindian ancestry. Their longitudinal study showed reduced WMH progression among those consuming 8.8 or more servings of oily fish per week compared to those with lower intake [165].

In summary, these studies collectively highlight the potential cognitive and neuroprotective benefits of ω-3 PUFAs and related supplements in aging populations, emphasizing their role in supporting cognitive health and potentially mitigating age-related cognitive decline (Table 5).

### 3.3. Epidemiological Studies

Epidemiological studies have explored the relationship between dietary intake of ω-3 PUFA and cognitive health (Table 6). Seafood serves as a significant dietary source of long-chain ω-3 PUFAs, which have been associated with enhanced cognitive function in middle-aged and older adults. A cross-sectional study by Benchao Li and colleagues highlighted that higher intake of long-chain ω-3 PUFA and elevated blood mercury levels were associated with improved cognitive performance among middle-aged and older Chinese adults [167]. Contrary to expectations, Amman E.M., et al. found no significant association between levels of DHA and EPA and cognitive decline in elderly women without dementia [168]. Their study indicated that women with both the highest and lowest levels of these fatty acids experienced similar rates of cognitive change over time. In another population-based cohort study, Nozaki, Shoko et al. explored the association between dietary fish consumption and midlife intake of ω-3 PUFAs with the prevention of dementia later in life [169]. They highlighted that higher consumption of fish, particularly those rich in DPA, DHA, and EPA, was associated with a reduced risk of dementia, though ALA did not show similar benefits. Conversely, a long-term study spanning 9.6 years in the Netherlands found no conclusive evidence linking fish consumption or ω-3 PUFAs intake with the risk of dementia [170]. Lastly, Gustafson D.R. et al. conducted an observational study revealing that increased dietary intake of DHA and EPA was associated with a decreased risk of AD among elderly individuals aged 65 years and older [171]. Their results are consistent with earlier epidemiological research showing the unique role of EPA and DHA, compared to overall PUFA intake or other long-chain fatty acids, in reducing the incidence of dementia associated with aging.

## 4. Challenges in Omega-3 Supplementation and Potential Solutions

Despite the extensive evidence supporting the benefits of ω-3 PUFAs, particularly DHA and EPA, on bone health and aging, several challenges remain in their effective supplementation and utilization. Clinical results have been inconsistent, with some studies showing positive outcomes, while others report no effect or even negative results. These discrepancies are largely attributed to the bioavailability of DHA and EPA. Bioavailability refers to the proportion of an administered drug dose that reaches the bloodstream as the active ingredient, making it available to the body to produce a therapeutic effect [173]. Bioavailability is determined by several factors, such as the chemical and physical properties of the drug, the method of administration, interactions with other substances, absorption, liver metabolism, and excretion [174,175]. The bioavailability of active pharmaceutical ingredients (API) refers to how much of the dose enters the bloodstream, directly impacting its effectiveness in treatment [176].The beneficial effects of omega-3 supplements are limited by their very low water solubility and poor oral bioavailability. The hydrophobic nature of DHA makes it difficult to dissolve in the aqueous environment of the gastrointestinal tract. This low solubility prevents its effective absorption by the intestinal tract, resulting in reduced DHA levels in the bloodstream. Inadequate absorption can lead to the excretion of a large portion of DHA even when supplements are taken. Another factor influencing the bioavailability of omega-3 fatty acids is the structural arrangement of long-chain ω-3 PUFAs in triglyceride molecules. ω-3 PUFAs, including DHA, are primarily esterified at the sn-2 position of triglycerides, which is less favourable for lipase hydrolysis, leading to reduced absorption and bioavailability in the body [177]. Seal oil, another marine source of long-chain ω-3 PUFAs, has a unique structure where ω-3 PUFAs are esterified at the sn-1 and sn-3 positions [178,179]. This enhances lipase hydrolysis, improving absorption and bioavailability compared to fish oil [180]. This structural difference highlights the challenge of DHA’s lower bioavailability in fish oil, emphasizing the need for more efficient delivery systems to enhance absorption and therapeutic effects. DHA needs to be transported and metabolized after absorption, but deficiencies in these processes can reduce the amount of DHA available for biological purposes [181].

The poor bioavailability of these fatty acids necessitates high doses to achieve therapeutic effects, which can be impractical and less effective in clinical settings. In most clinical studies, the dosages of DHA and EPA used are relatively high, ranging from 0.2 to 2.5 g [182]. However, long-term use of these high doses can lead to severe side effects and is not always practical for sustained use. These doses often result in adverse effects like a persistent fishy aftertaste, which can discourage patient compliance. Moreover, gastrointestinal issues such as burping may occur due to the oxidative breakdown of the fatty acids in the digestive system [183]. These challenges not only affect patient satisfaction but also limit the clinical effectiveness of high-dose treatments, as the body may struggle to absorb them efficiently.

In addition to bioavailability challenges, incorporating ω-3 PUFAs into drugs and supplements is hindered by their susceptibility to oxidative degradation, particularly EPA and DHA, which contain multiple double bonds and are highly prone to lipid oxidation [184]. Taken out of the biological context, the polyunsaturated nature of these substances makes them highly susceptible to oxidation by molecular oxygen (O2) present in the air [185]. This oxidation not only reduces shelf-life but also impacts consumer acceptability, functionality, nutritional value, and safety of PUFA-rich foods and fish oil supplements [186]. When fish oils oxidize, they produce unstable intermediates like free radicals and hydro-peroxides, which decompose into aldehydes and ketones. These compounds degrade nutritional quality and contribute to off-flavours and health risks [187]. The detection of fatty acid oxidation products in food, particularly aldehydes, has been associated with processes such as ageing, mutagenesis, and carcinogenesis [188]. Furthermore, frequent ingestion of the by-products of lipid oxidation over a long period of time can exhibit toxicity and cause long-term health problems [189]. To address the stabilization challenges of ω-3 PUFAs, strategies include encapsulation with nanoparticles to reduce oxidation and enhance stability, as well as the use of specialized pro-resolving mediators (SPMs) derived from DHA and EPA, which are more bioavailable and effective at lower dosages.

### 4.1. Specialized Pro-Resolving Mediators

Recent research highlights the potential of DHA and EPA metabolites as a promising solution to these challenges (Table 7). DHA and EPA can be bio-actively transformed in vivo by enzymes into new lipid metabolites called specialized pro-resolving mediators SPM, such as resolvins (RvD1, RvD2, RvE1), neuroprotectin D1 (NPD1), and maresin 1 (MaR1) [190]. These metabolites are more bioavailable, requiring lower dosages to achieve similar or superior therapeutic effects compared to their parent compounds [191]. SPMs have stronger biological activity and better absorption, which may help overcome the shortcomings of ω-3 PUFAs supplementation and provide a more effective way to obtain the health benefits of them [192]. This increased bioavailability addresses a significant limitation in the current use of omega-3 supplements for bone health and aging.

SPMs have strong anti-inflammatory and pro-resolution effects, offering potential therapeutic benefits for bone health [193]. Bone loss can occur from inflammatory bone conditions that can be harmful to human health. Numerous studies have demonstrated that low dosages (in the nanogram range) of SPMs administered peripherally, spinally, or orally effectively alleviate inflammatory pain [194,195,196]. In contrast, DHA and EPA typically need to be administered in much higher doses, often exceeding 0.25 g/d, to achieve similar benefits for bone health.

Norling et al. observed significant reductions in joint inflammation in C57Bl/6 mice with inflammatory arthritis following administration of 100 ng/day of the stable epimer 17R-RvD1 [193]. Using lipid mediator metabololipidomics, RvD1 was detected in both mice arthritic paws and human RA synovial fluids. This treatment decreased leukocyte infiltration, alleviated arthritic symptoms, and preserved cartilage, demonstrating its therapeutic potential. 

In studies by Jiang et al., RvD1 administration via collagen 3D nanopore scaffold (COL) and Pluronic F127 hydrogel (F127) in rat calvarial defects showed enhanced osteoid formation and vascularization, suggesting its potential for bone healing [197]. Similarly, in a rat femoral defect model, embedding resolvin D1 (RvD1) in chitosan (Ch) porous 3D scaffolds over 2 months improved bone regeneration, structure, trabecular thickness, and the collagen type I/Coll I/Coll III ratio. The study demonstrated that low dosage of RvD1 improves bone regeneration by positively regulating the inflammatory response, underlining its potential in advanced regenerative medicine approaches [198].

The antioxidant properties of novel imidazole-derived RvD1 analogues explored by Kariminezhad et al. suggest their potential for treating oxidative stress-related diseases such as OA. These analogues have shown promise in enhancing joint mobility by prolonging the lifetime of hyaluronic acid (HA) in the knee [199]. In vivo studies by Sara Alrumaih et al. demonstrated that combining Resolvin E1 (RvE1) with a bovine bone graft significantly promoted bone regeneration in critical-size defects in Wistar rats’ calvaria compared to using either RvE1 or the graft alone [200]. This underscores RvE1’s efficacy, particularly when integrated with bone-grafting techniques, in facilitating bone repair.

The impact of SPMs on neuroinflammation and cognitive function has also been a subject of extensive research. Previous research identified impaired PI3K/AKT signalling in AD patients [201]. In a model study, MaR1 treatment up-regulated PI3K/AKT signalling, reduced pro-inflammatory cytokines (TNF-α, IL-6), increased anti-inflammatory cytokines (IL-2, IL-10), and improved cognitive function [202]. Further supporting these observations, intranasal administration of a mixture of specialized pro-resolving mediators (SPMs), including RvE1, RvD1, RvD2, MaR1, and NPD1, in mice led to improved memory, restoration of gamma oscillations, and a significant reduction in microglial activation [203].

These findings collectively highlight the therapeutic potential of SPMs. Notably, all the studies mentioned demonstrate the effectiveness of SPMs at very low doses, typically less than 100 ng or 100 micro molar. This contrasts with DHA and EPA, which often require higher doses to achieve comparable effects. The ability of SPMs to deliver positive outcomes at such low concentrations underscores their efficacy while minimizing potential side effects, thereby suggesting promising avenues for clinical applications and future research directions.

**Table 7:** Summary of studies from 2016 to 2024 highlight the therapeutic benefits of SPMs at very low concentrations or dosages, demonstrating significant efficacy in various inflammatory, bone health, and cognitive conditions.

Reference	Sample Size (n) and Population	Treatment	Duration	Results	Overall Outcome
Norling, LV et al., (2016) [193]	Male 12-week-old, C57Bl/6 mice (30 g) with induced Rheumatoid arthritis (RA)	Stable epimer 17R-RvD1 (100 ng/day)	32 days	- 17-RvD1 reduced joint inflammation and destruction, enhanced chondroprotection through increased expression of cartilage matrix synthesis genes in RA patients. - 17R-RvD1 significantly reduced arthritis severity, cachexia, hind-paw edema, and leukocyte infiltration, shortened the remission interval, and increased levels of SPMs.	Positive
Funaki, Y et al., 2018 [204]	RAW264.7 cells and Mouse calvarial cells (MC3T3-E1)	100 nM RvE1	7 days	- RvE1 suppressed osteoclastogenesis and bone resorption by inhibiting RANKL-induced NFATc1 and c-fos in osteoclasts and IL-17-induced RANKL in osteoblasts. - Co-treatment with 100-nM RvE1 reduced resorption pit area, evidenced by decreased TRAP-positive cells, increased OPG expression, and a favorable RANKL/OPG ratio, enhancing bone formation.	Positive
Vasconcelos, DP et al., 2018 [198]	3-month-old male Wistar rats with femoral defect	- Ch + RvD1 scaffolds with 30 μL of RvD1 solution - Chitosan scaffolds without RvD1 (control)	2 months	- RvD1 enhanced bone tissue repair, shown by increased collagen type I fibers, a higher Coll I/Coll III ratio, greater trabecular thickness, and a higher bone volume in the Ch+RvD1 group compared to the Ch group.	Positive
Yin, P et al., 2019 [202]	C57BL/6 mice (male, 3–4 months old, weight 26–31 g)	0.01 µg/µL MaR1	7 days	- MaR1 improved cognitive decline by reducing pro-inflammatory cytokines (TNF-α, IL-6, MCP-1) and increasing anti-inflammatory cytokines (IL-2, IL-10) levels.	Positive
Emre, C et al., 2022 [203]	C57BL/6 J wild-type (WT) mice	40 ng per LM of RvE1, RvD1, RvD2, MaR1, and NPD1	9 weeks	- SPMs improved brain health and cognitive performance by reducing microglial activation, which in turn led to enhanced memory function and the restoration of normal brain wave patterns (gamma oscillation) in the brain.	Positive
Jiang, X et al., 2022 [197]	Female Sprague-Dawley (SD) rats (180–200 g) with induced calvarial defects	Implanted collagen 3D nanopore scaffold (COL) and Pluronic F127 hydrogel (F127) with or without RvD1 - RvD1-COL-F127 group (Added 5 ng/μL RvD1 to F127 solution) - COL-F127 group (control) - Subcutaneous RvD1: 100 ng every 7 days post-implantation.	8 weeks	- RvD1 enhanced bone formation and vascularization while reducing bone resorption in rat calvarial defects. - It decreased TRAP-positive cells and inflammatory cytokines (IL-1β and TNF-α), lowered RANKL expression, and increased osteoid-like tissue, blood vessel count, ALP, and VEGF in the RvD1-COL-F127 group.	Positive
Sara Alrumaih et al., 2023 [200]	Healthy female Wistar rats 250–300 g with induced critical-size calvarial defect	- Negative control with no treatment - Positive control with bovine xenograft - RvE1 alone 100 ng - RvE1 100 ng + bovine xenograft	12 weeks	- RvE1 improved bone mineral density and formation in the RvE1 + bovine xenograft group, which showed the highest ALP and OPN gene expression, as well as the highest mean NFB.	Positive
Al Zahrani, S et al., 2024 [205]	Human bone marrow MSCs	100 nM concentration of RvE1 and MaR1	14 days	- Both RvE1 and MaR1 promoted osteogenic differentiation of hBMMSCs, enhancing bone formation by increasing calcified deposits and ALP activity.	Positive	

Abbreviations: ALOX15: arachidonate 15-lipoxygenase, ALP: alkaline phosphatase, Ch: chitosan, Coll I/Coll III: Collagen type I and collagen type III, Il1b: Interleukin 1 beta, LM: lipid mediator, Ly6g: Lymphocyte antigen 6 complex locus G6D, MaR1: Maresin 1, MMP-9: matrix metalloproteinase-9, MSCs: mesenchymal stem cells, NFATc1: nuclear factor of activated T cells c1, NFB: newly formed bone volume, NPD1: Neuroprotectin D1, OPG: osteoprotegerin, OPN: osteopontin, PDx: protectin Dx, PGE2: Prostaglandin E2, RANKL: receptor activator of nuclear factor-κB ligand, RvD1: Resolvin D1, RvE1:resolvin E1, SPM: specialized pro-resolving mediator, TNF-α: tumor necrosis factor-α, TRAP: tartrate-resistant acid phosphatase, µCT: Micro-computed tomography, VEGF: vascular endothelial growth factor.

### 4.2. Enhanced Delivery of Omega-3 Fatty Acids through Nano Encapsulation

**Figure 4:**
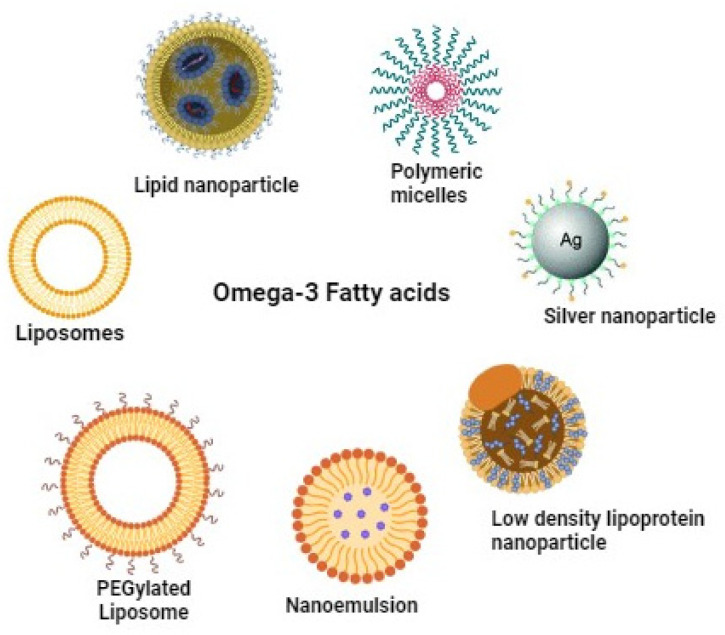
Examples of nanoparticles used to encapsulate, protect, and deliver ω-3 PUFAs.

Recent advancements in oil-in-water emulsification and encapsulation offer promising strategies to enhance the stability of fish oil rich in EPA and DHA against oxidative damage from free radicals, oxygen, and metal ions [206,207,208]. Nano encapsulation involves encapsulating bioactive compounds in nanoparticles (NPs) to create stable formulations (Figure 4). The utilization of NPs in the delivery of ω-3 PUFAs represents a cutting-edge approach in Nano biotechnology, utilizing nanometric materials sized between 1 and 100 nm to enhance the bioavailability and stability of these essential nutrients [209]. The unique properties of NPs, such as their solubility, high surface-to-volume ratio, and strength, are instrumental in enhancing drug-delivery efficiency and effectiveness. [210]. Thus, Nano encapsulation technology enhances the poor solubility of omega-3 fish oil by facilitating the formation of a micelle network or by minimizing interactions with other substances [211]. This approach prevents colour changes, masks undesirable flavours, enables controlled release of minerals, ensures product stability during production and storage, and improves product characteristics [212]. Additionally, the controlled and sustained release of contents from nonencapsulated bioactive substances improves their solubility in water and increases their availability for biological activities [213]. Research also indicates that the size of nanoemulsion droplets positively influences the bio-accessibility of encapsulated bioactive chemicals, enhancing the digestibility of the lipid phase [214]. Therapeutic and diagnostic nanoparticles fall into two main categories: organic (e.g., liposomes, micelles, polymers) and inorganic (e.g., gold, iron oxide). Organic nanoparticles are employed in diagnostic procedures, imaging techniques, and photothermal therapy [215]. Inorganic nanoparticles are significant in preclinical studies, including cancer imaging and treatment [216], drug delivery, magnetic targeting, and treating anaemia. Nanoparticle encapsulation of ω-3 PUFAs shows promise in treating various conditions such as improving cognition, reducing inflammation, managing diabetes, and treating brain injuries, as outlined in Table 8.

Targeting inflammation through nanoparticle delivery of ω-3 PUFAs aims to reduce symptoms associated with prevalent and serious pathologies, particularly affecting bone health and neurodegeneration. In a study by Alaarg A et al., DHA was integrated into polyethylene glycol (PEG)-coated liposomes and assessed for anti-inflammatory effects in various immune cells [217]. These long-circulating PEGylated liposomes can gather in inflamed tissues and release their PUFA cargo into macrophages and other immune cells, demonstrating potential benefits in treating inflammatory disorders. In another study, encapsulated ω-3 PUFA in novel Nanotex superparamagnetic nanoparticles showed promising anti-inflammatory effects for image-guided drug delivery. They exhibited significant activity against colonic inflammation and demonstrated important anti-tumoral effects against glioma, specifically slowing proliferation and inducing remission in glioma C6 cells [218].

Recently, oil-in-water nanoemulsions have become crucial in the food industry for encapsulating lipophilic bioactive compounds effectively [219]. Nunes R et al. explored using protein-based nanoemulsions to stabilize ω-3 PUFAs in water [220]. They employed lactoferrin (Lf) as an emulsifier at varying concentrations, finding that higher Lf levels resulted in smaller nanoemulsion sizes. Lactoferrin (Lf), a glycoprotein from the transferrin family, exhibits antimicrobial, antiviral, and antioxidant properties, enhancing the chemical stability of emulsions when added as an additive or deposited at the oil-in-water interface [221]. Results demonstrated that nanoemulsions remained stable at 4 °C for 69 days when encapsulated in Lactoferrin (Lf), maintaining structural integrity. These nanoemulsions exhibited antioxidant properties by scavenging free radicals. Nanoemulsions containing 2% (*w*/*w*) Lf and 12.5 μg/mL of omega-3 were non-cytotoxic to Caco-2 cells, indicating the safety of the encapsulation method. A recent study used PLGA and chitosan nanoparticles (PCSDNP) to deliver DHA, achieving better stability and controlled release [222]. Studies have shown that chitosan ensures biological safety and protects active substances during digestion [223], while PLGA enhances the solubility and stability of hydrophobic substances [224].A study by Mulik RS et al. found that LDL DHA administration, combined with pulsed focused ultrasound, doubled DHA levels and increased Resolvin D1 in targeted brain areas. This method allows localized DHA delivery to the brain and could be useful in treating acute brain injuries [225].

The Omacor^®^ soft capsule is a well-known ω-3 ethyl ester product prescribed at high doses (2 to 4 g once or twice daily) [226]. However, its large capsule size poses a challenge for patient compliance due to difficulty in swallowing, potentially affecting treatment effectiveness. In a randomized crossover study, a novel liquid crystalline nanoparticle-based formulation called IMD-Omega soft capsule significantly improved EPA and DHA bioavailability: EPA by 110% and DHA by 134% over 72 h compared to Omacor^®^ soft capsule [227]. Serini, S. et al., conducted a study to investigate the potential benefits of DHA encapsulated in resveratrol-based solid lipid nanoparticles (DHA-RV-SLNs) on skin health. They found that DHA-RV-SLNs significantly enhanced the protective effects of DHA against cytotoxicity induced by surfactants in human keratinocytes, suggesting a potential role in protecting the skin from environmental irritants and inflammatory processes [228]. Metal nanoparticles like gold (AuNPs), silver (AgNPs), and zinc oxide (ZnONPs) can effectively deliver medical drugs to specific organs without side effects [229,230,231,232]. Studies demonstrate the antidiabetic effects of ZnONPs and AgNPs in controlling diabetes mellitus in rats [233]. Recent research using nanoprecipitation to prepare DHA/AgNPs highlights their potential to alleviate diabetic complications and improve endothelial dysfunction in experimental diabetes [234].

**Table 8:** Summary of studies from 2015 to 2023 that used nanoparticle-encapsulated omega-3 fatty acids to improve cognition, treat brain injuries, and reduce inflammation.

Reference	Nanoparticle Type	Loaded Drug/ Compound/ Molecule	Nanoparticles Characteristics	Findings
Calle, D et al., 2015 [218]	liposomes containing the superparamagnetic nanoparticle Nanotex	ω-3 PUFA ethyl esther	Particle size: 200 nm	Demonstrated significant anti-inflammatory effects against colonic inflammation and important anti-tumoral effects against glioma.
Alaarg, A et al., 2016 [217]	polyethylene glycol (PEG) liposomes	DHA	Particle size: 99 ± 16 nm encapsulation efficiency = 81.35 ± 3.24%	ω-liposomes represent a promising nanonutraceutical formulation with potential benefits for treating inflammatory disorders.
Mulik, R.S et al., 2016 [225]	Low-density lipoprotein (LDL) nanoparticles	DHA	Particle size = 22.4 ± 0.71 nm	LDL DHA administration doubled DHA and Resolvin D1 levels in targeted brain areas, enabling localized delivery for treating acute brain injuries.
Hussein, JS et al., 2019 [234]	Silver nanoparticles	DHA	Particle size: 24 nm encapsulation efficiency = 97.67%	DHA combined with silver nanoparticles (AgNPs) shows significant potential in alleviating diabetic complications and improving endothelial dysfunction in experimental diabetes.
Serini, S et al., 2019 [228]	Encapsulated in Resveratrol-Based Solid Lipid Nanoparticles (RV-SLNs)	DHA	Particle size: 139.27 nm	By encapsulating DHA in solid lipid nanoparticles, the DHA-RV-SLNs could enhance the protective effects of DHA against cytotoxic actions of surfactants in human keratinocytes.
Nunes, R et al., 2020 [220]	Nanoemulsions utilizing Lactoferrin (Lf) as an emulsifier	DHA and EPA	Particle size < 200 nm Encapsulation efficiency = > 99%	Resulted in improved stability, controlled release profiles at different pH levels, antioxidant properties, and non-cytotoxicity to Caco-2 cells.
Liu, E et al., 2021 [222]	nanoparticle with PLGA and chitosan (PCSDNP)	DHA	Particle size = 256 nm encapsulation efficiency = 87%	PCSNP enhances the stability of DHA and protects it from oxidation or degradation in the gastrointestinal tract. It also provide a slow-release effect, improving the digestion and absorption of DHA.
Kang, KM et al., 2023 [227]	liquid crystalline nanoparticle-based formulation called IMD-Omega soft capsule	DHA and EPA	Particle size: < 154 ± 61 nm	The IMD-Omega soft capsule showed a substantial 110% increase in EPA bioavailability and an impressive 134% increase in DHA bioavailability over 72 h.

Abbreviations: DHA: docosahexaenoic acid, PLGA: Poly-D, L-lactide-co-glycolide.

## 5. Conclusions

In conclusion, this review underscores the potential benefits of ω-3 PUFAs, particularly DHA and EPA, in mitigating significant health challenges such as osteoporosis and age-related neurodegenerative diseases including cognitive decline, dementia, and Alzheimer’s disease. Epidemiological studies suggest a protective role of higher omega-3 intake against bone mineral density (BMD) loss, as populations with elevated consumption exhibit lower osteoporosis rates. Consistently, preclinical research demonstrates that omega-3 supplementation enhances bone mass and strength. However, clinical trials present a more complex picture, with conflicting results regarding the direct impact of ω-3 PUFAs on BMD across diverse populations.

In the context of aging-related neurodegenerative diseases, preclinical studies highlight the neuroprotective effects of DHA and EPA in reducing oxidative stress and inflammation, enhancing neuroplasticity, and influencing neurotransmitter pathways. Epidemiological evidence generally supports a protective role of omega-3s against age-related neurodegenerative disease with higher dietary intake. However, clinical studies yield mixed results on cognitive benefits in older adults, revealing variability in study design, dosage, and participant characteristics.

Significant variations between studies are evident, with dramatic beneficial effects observed in animal models not consistently mirrored in human studies. These discrepancies can be attributed to differences in ω-3 PUFAs dosages between animal and clinical studies, as well as variations in the source of omega-3s, whether from supplements or diet. The poor bioavailability of DHA and EPA, influenced by low water solubility, poor absorption, and susceptibility to oxidative degradation, further complicates their clinical efficacy. High doses required for therapeutic effects are often impractical and may lead to severe side effects.

Despite these challenges, this review provides two promising solutions: Use of specialized pro-resolving mediators (SPMs) and nanoparticle encapsulation. As discussed in this article, SPMs, which are metabolites of DHA and EPA, offer enhanced bioavailability and stronger biological activity at significantly lower doses compared to their parent compounds. SPMs such as resolvins, Maresin 1, and protectins exhibit potent effects in reducing inflammation, promoting bone health, and protecting against neurodegenerative diseases. Studies have shown that SPMs can achieve these benefits at very low doses (less than 100 ng), making them a viable option for overcoming the limitations of traditional omega-3 supplementation. Additionally, nanoparticle encapsulation of ω-3 PUFAs represents an effective method to increase their bioavailability, reduce oxidation, and achieve controlled release. Various studies highlighted in this article have demonstrated the use of liposomes, lipoproteins, metal nanoparticles, nanoemulsion, liquid crystalline nanoparticles, chitosan nanoparticles, and resveratrol-based solid lipid nanoparticles to effectively enhance muscle and bone strength, reduce inflammation, improve cognition, and aid recovery from brain injuries.

This review discussed the role of ω-3 PUFAs in bone health and aging, highlighting both the promising results and the challenges observed in clinical studies. We also described the benefits of SPMs and nanoparticle encapsulation, emphasizing their potential to improve the clinical efficacy of ω-3 PUFAs.

Overall, while the potential benefits of ω-3 PUFAs are promising, further research is essential to improve their bioavailability and determine optimal therapeutic dosages for effective clinical application.

## Figures and Tables

**Table 1:** Summary of outcomes of pre-clinical studies published between 2013 and 2023 on bone health utilizing diets rich in ω-3 FAs.

Reference	Model Type In Vitro/In Vivo	Treatment (Consisting of ɷ-3 FA)	Duration	Results	Overall Outcome
Casado-Díaz, A et al., 2013 [61]	Human MSC	- Control = ethanol - Omega 6 fatty acids = 20 μM and 40 μM of AA - Omega 3 fatty acids = 20 μM and 40 μM of DHA, EPA	21 days	- ω-3 FAs reduced bone loss by increasing the OPG/RANKL ratio in osteoblasts and decreasing adipogenesis in MSCs. DHA and EPA (20 μM) enhanced bone health by increasing osteogenic markers (Runx2, OSX, ALP) and not affecting adipogenesis marker PPARγ2, supporting osteoblastogenesis without promoting fat cell development.	Positive
Nakanishi, A et al., 2013 [86]	Ovariectomized or sham-operated (sham) Female Wistar/ST rats (9 weeks old)	- Control = Corn oil - Fish oil (FO) = DHA -9.2 g/100 g, EPA -12.8 g/100 g	2 weeks	- ω-3 FAs treatment decreased mRNA levels of M-CSF, PU.1, MITF, RANK, RANKL, and serum TNFα, IL-6, and PGE2, promoting bone health by reducing ovariectomy-stimulated osteoclastogenesis.	Positive
Abou-Saleh et al., 2019 [87]	11-months-old C57BL/6 female mice	- 4% SFO safflower oil (70–80% ω-6 fatty acids, mainly LA) - 1% CFO concentrated Fish oil [46.5% EPA and 37.5% DHA] - 4% CFO - 4% regular-fish oil FO - Untreated control group with standard lab chow diet	12 months	4% CFO Group - The treatment exhibited the highest BMD across all bone regions compared to other groups. It prevented aging-associated bone loss by modulating osteoclastogenesis and bone resorption, resulting in reduced levels of bone resorption markers RANKL and TRAP5b. 1% CFO Group - The treatment increased lumbar, tibial, and femoral BMD while decreasing the NF-κB, JNK, and p38 MAPK signaling pathways, which are associated with bone resorption. 4% Regular Fish Oil Group - The treatment moderately increased BMD in various bone regions compared to the 4% SFO group, enhancing reducing bone resorption, indicated by lower serum RANKL and TRAP5b levels.	Positive
Anez-Bustillos et al., 2019 [88]	3-weeks-old male and female C57BL/6J mice	- SOY diet - DHA/ARA diet in 20:1 ratio - DHA diet	9 weeks	- DHA increased total area (TA) and bone area (BA) compared to SOY and DHA/ARA, but had no major effect on cortical or trabecular bone, showing no significant impact on bone metabolism.	Neutral
Bani Hassan, E et al., 2019 [68]	1-month-old female senescence accelerated mouse prone 8 (SAMP8) mice	- Sunflower oil-based Diet (Total ω-3 PUFA = 2.29%) - Borage oil enriched Diet (Total ω-3 PUFA = 2.35%) - FO enriched diet (Total ω-3 PUFA = 11.60%)	10 months	- The FO group increased bone-volume fraction and decreased MAT%, which was positively associated with bone health by reducing age-related bone loss and hematopoietic bone marrow loss (HBM) through limited MAT expansion.	Positive
Elbahnasawy, AS et al., 2019 [89]	12-week-old male Sprague Dawley rats	- Control = sunflower oil - Prednisolone control (10 mg/kg prednisolone per daily) - Prednisolone+ Soybean oil diet (7% SBO) - Prednisolone + Flaxseed oil diet (7%) - Prednisolone + Fish oil diet (7% FO with EPA = 19.12% and DHA = 21.08%)	3 weeks	- Fish oil increased plasma calcium levels and reduced oxidative stress and inflammatory markers. It suppressed bone resorption, as indicated by decreased CTX levels, increased BMD, and normal histological results compared to controls, effectively mitigating GC-induced osteoporosis.	Positive
Xie, Y et al., 2019 [90]	12-week-old male Sprague Dawley rats	Anterior cruciate ligament transection ACLT-operated and treated with 1 mg/kg DHA per day	2 months	- DHA-treated rats experienced less bone-mass loss, with reduced TRAP, RANKL, CD31, and endomucin levels in the osteochondral unit, indicating decreased bone resorption. DHA also restrains bone remodeling and vessel formation, potentially protecting cartilage.	Positive
Xie, Y et al., 2019 [90]	Monocyte-derived cell line RAW264.7	Three groups: sham, vehicle-treated (RANKL), and DHA treated (RANKL + DHA).	2 months	- In DHA-treated cells, TRAP-stained cells, bone resorption pits, and mRNA levels of TRAP, CTSK, MITF, and NFATC1 were lower, indicating DHA inhibits osteoclast differentiation and bone resorption. DHA-treated RAW264.7 cells exhibited fewer resorption pits than RANKL-only exposed cells, and DHA also attenuated cartilage degeneration.	Positive
Dai, Y et al., 2021 [77]	8-week-old female C57BL/6J healthy and osteoarthritis mice	- ω-6 FA with 1.57% AKO [Antarctic krill oil] - ω-3 FAs with 39.66% AKO Containing DHA + EPA = 37.81% - n-6/n-3 ratio checked = 20:1, 6:1, 1:1	10 weeks	- Low n-6/n-3 PUFA ratios (1:1 to 6:1) improved cartilage structure, reduced polysaccharide loss, and decreased NF-κB signaling via GPR120 activation, thereby reducing inflammation and supporting bone health. These diets, including AKO rich in DHA and EPA, reduced cartilage degeneration, which can enhance bone formation and decrease resorption.	Positive
Zhang, T et al., 2021 [81]	8-week-old female C57BL/6 OVX mice [OP+OA Mice Model]	n-6/n-3 PUFAs - 20:1 non- FO - 20:1 FO (700 mg FO/kg) - 20:1 FO-High (1400 mg FO/kg) - 6:1 non-FO - 6:1 FO-Low (700 mg FO/kg) - 6:1 FO-High (700 mg FO/kg)	12 weeks	- High dose FO improved bone and cartilage health in the osteoporotic OA model, even in a high n-6/n-3 PUFA diet. 20:1 FO-H improved bone microarchitecture, GPR120 expression, and BMD while enhancing osteogenesis. It decreased bone resorption by lowering TNF-α, PGE2, IL-1β, NFκB, and NLRP3 expression in cartilage. 6:1 FO-H diet performed best for bone improvement, reducing cartilage degradation and restoring cartilage area and thickness.	Positive
Cifuentes-Mendiola, SE et al., 2022 [84]	Human osteoblast cell line hFOB 1.19	DHA (10 and 20 µM)	21 days	- DHA enhanced bone mineral matrix quality and reduced oxidative stress induced by 24 mM glucose (HG). Osteoblasts cultured in HG and treated with DHA exhibited increased collagen type 1 (Col1) scaffolds, elevated OCN and BSP-II expression, increased NRF2 mRNA, and reduced ROS production, promoting bone health.	Positive
Tompkins, YH et al., 2022 [70]	Ross 708 Broiler breederhen	- Control = 2.3% of soybean oil (SO) Intervention group (IG) = 2.3% FO [18% EPA and 12% DHA]	28 days	- Maternal fish oil (FO) diet rich in ω-3 PUFAs positively impacted fat mass and skeletal integrity in broiler offspring. It increased bone mineral content (BMC), total volume, and bone surface (BS) in the tibia of 18-day-old embryos, indicating enhanced bone formation. Additionally, it reduced pore volume and levels of adipogenic transcription factors PPARγ, FABP4, and C/EBPβ, suggesting a reduction in adipogenesis.	Positive
Zhu, Y et al., 2022 [83]	3-month-old female pure Wistar rats	- OVX - E2 (OVX + high-dose estrogen) - nH (OVX + high-dose THLs) - nL (OVX + low-dose THLs) - Tilapia nilotica fish head lipids THLs containing neutral lipids (NL, 77.84%), phospholipids (PL, 11.86%), and glycolipids (GL, 6.47%)	1 week	- Both high- and low-dose THL groups improved trabecular microstructure by enhancing bone formation (increased BV/TV, cortical density, and Tb.N) and reducing bone resorption (decreased Tb.Sp and SMI), supporting bone health. - Treatment with THLs restored the OPG/RANKL ratio and reduced bone resorption markers CTX-1, Cath-K, and MMP-9, thus improving bone health through decreased bone resorption.	Positive
Ku, SK et al., 2023 [76]	Rat model of knee Osteoarthritis (OA)	Orally administered KO at 200, 100, and 50 mg/kg (KO200, KO100, and KO50	8 weeks	- Both KO200 and KO100 groups exhibited increased BMD, reduced joint capsule thickness, and enhanced strength of femoral and tibial articular cartilage compared to the OA control. Additionally, oral KO doses promoted chondrogenic gene expression (type 2 collagen, aggrecan, Sox9), while decreasing inflammation markers (5-lipoxygenase, PGE2) and ECM-degrading enzymes (MMP-2, MMP-9) in cartilage and synovium, indicating improved joint health and cartilage preservation.	Positive

Abbreviations: AA/ARA: arachidonic fatty acid, ACLT: anterior cruciate ligament transection, ALP: alkaline phosphatase, BGLAP: bone gamma-carboxyglutamic acid-containing protein, BMD: Bone mineral density, BSP: bone sialoprotein, CTSK: Cathepsin K, DHA: docosahexaenoic acid, EPA: eicosapentaenoic acid, FABP4: Fatty acid binding protein 4, GPD1: glycerol-3-phosphate dehydrogenase, IL-6: interleukin-6, KO: Krill oil, LA: linoleic acid, MAT: Marrow adipose tissue, MCS-F: macrophage colony-stimulating factor, MITF: microphthalmia-associated transcription factor, MMP-9: matrix metalloproteinase-9, MSC: mesenchymal stem cell, NFATC1: nuclear factor of activated T-cells cytoplasmic, NF-κB: Nuclear factor kappa-light-chain-enhancer of activated B cells, NLRP3: nucleotide-binding domain, leucine-rich–containing family, pyrin domain–containing-3, NRF2: nuclear factor erythroid 2-related factor-2, OC/OCN: osteocalcin, OPG: osteoprotegerin, OPN: osteopontin, OSX: osterix, OVX: Ovariectomized, PGE2: prostaglandin E2, RANK: receptor for activation of NFκB, RANKL: receptor activator of nuclear factor-κB ligand, ROS: reactive oxygen species, runx2: runt-related transcription factor 2, SMI: structural model index, SPP1: secreted phosphoprotein, SREBP1: sterol regulatory element-binding transcription factor 1, Tb.n: trabecular number, Tb.sp: trabecular bone separation, TNFα: tumor necrosis factor α, TRAP: Tartrate-Resistant Acid Phosphatase.

**Table 2:** Summary of outcomes of clinical studies published between 2005 and 2024 on bone health utilizing diets rich in ω-3.

Reference	Sample Size (n) and Population	Age/Mean ± SD Age	Treatment	Duration	Results	Overall Outcome
Trebble, TM et al., 2005 [100]	61 Crohn’s patients	40–45	Fish oil supplement = 2.7 g/d EPA and DHA, (n = 31) Placebo = olive oil, containing the MUFA oleic acid (n = 30)	24 weeks	- High intakes of EPA and DHA from fish oil do not appear to impact bone health in individuals with Crohn’s disease, as they do not influence bone-turnover indices, evidenced by no differences in bone resorption marker Deoxypyridinoline (DPD), bone formation marker Osteocalcin levels, or the DPD/Osteocalcin ratio.	Neutral
Martin-Bautista, E et al., 2010 [109]	72 Hyperlipidaemia patients	35–65 years	Control [group C] = 2.05 g oleic acid per 500 mL semi skimmed milk, (n = 33) Group E -fortified supplement group = 5.17 g oleic acid, 0.14 g DHA, and 0.20 g EPA per 500 mL, (n = 39)	12 months	- Fortified milk increased bone markers, including plasma OPG, OPG/RANKL ratio, and osteocalcin, thus improving bone health in hyperlipidaemic adults, while showing no change in intact parathormone (Ipth) and type I collagen carboxyl-terminal telopeptide (CTX) levels.	Positive
Appleton, KM et al., 2011 [99]	113 Mild–moderately depressed individuals	18–67 years	Intervention group = 1.48 g ω-3 PUFAs (0.63 g EPA + 0.85 g DHA)/d Placebo = olive oil	12 weeks	- Supplementation with ω-3 PUFAs did not prevent bone loss or reduce bone resorption, nor did it effect b-CTX levels (a marker of bone resorption) or overall bone resorption.	Neutral
Tartibian, B et al., 2011 [112]	79 healthy sedentary post-menopausal women	58–78 years	- (C) control (n = 18) - (E+S) exercise +supplement (n = 21) - (E) exercise only (n = 20) - (S) supplement only (n = 20) Supplement is 1000 mg of ω-3 PUFAs containing 180 mg EPA, 120 mg DHA.	24 weeks	- ω-3 FA improved bone health in post-menopausal osteoporosis by increasing BMD at the L2-L4 and femoral neck. It decreased inflammatory markers (TNF-α, PGE2, IL-6, and CTX) linked to bone resorption while increasing estrogen, calcitonin, and vitamin D and promoted overall skeletal integrity.	Positive
Vanlint, SJ et al., 2011 [113]	40 individuals with osteopenia (36 females, 4 males)	Mean age = 59.2 years	- Intervention group = 0.2 g DHA from algal oil in sunflower oil (two capsules/day) - Placebo = corn oil	12 months	- The addition of DHA (0.4 g/day) did not affect calcium and vitamin D levels in osteopenic individuals. Although CTX levels decreased by 10.5%, there was no improvement in bone health, as mean BMD values remained unchanged at the lumbar spine, total proximal femur, and neck of femur.	Neutral
Sabour, H et al., 2012 [114]	82 Osteoporotic patients with spinal cord injury (SCI), 1-year post injury.	≥18 years	MorDHA capsules = two capsules (435 g of DHA and 0.065 g of EPA per day) Control: Placebo Gelatin 1 g one capsule	4 months	- No significant changes were observed in bone-resorption markers, bone-formation markers, or pro-inflammatory cytokines in osteoporotic patients.	Neutral
Hutchins-Wiese, HL et al., 2014 [104]	38 Postmenopausal women on AI for ≥ 6 months for estrogen-positive breast cancer, continuing treatment for ≥ 1 year.	48–84 years Mean age = 62 years	Fish oil = seven capsules/day containing 4 g EPA + DHA (2.52 g EPA, 1.68 mg DHA) (n = 20) Placebo = seven capsules/day containing safflower oil (9% linoleic acid, 83% oleic acid) (n = 18)	3 months	- In the fish oil group, bone-resorption markers DPD and SCTX decreased, confirming a reduction in bone resorption, with a significant decrease observed in DPD levels.	Positive
Chen et al., 2015 [97]	202 Patients with knee osteoarthritis (49% female)	≥40 years Mean age = 61.0 ± 10.0 years	- High dose = 4.5 g EPA and DHA - Low dose = 0.45 g/day	2 years	- High-dose omega-3 fish oil did not alter bone loss in men and women with knee osteoarthritis, as indicated by no significant changes in BMD.	Neutral
Smith, GI et al., 2015 [115]	60 healthy adults	60–85years	- Four 1-gram capsules per day of omega-3 acid ethyl esters = 1.86 g EPA and 1.50 g DHA/day, equivalent to the ω-3 PUFAs content of 200–400 g freshwater fatty fish	6 months	- ω-3 PUFAs therapy enhanced thigh muscle volume, improved handgrip strength, increased one-repetition maximum (1-RM) muscle strength, and elevated average isokinetic power.	Positive
Fonolla-Joya, J et al., 2016 [108]	117 healthy postmenopausal women	50–70 years Mean age = 45 ± 7.7 year	Intervention group [IG] = 0.5 L/day of skimmed milk with hydrolysed lactose, enriched with 40 mg/100 mL EPA+DHA, 0.54 g/100 mL oleic acid, 0.5 g/100 mL soluble fiber, minerals, and vitamins (n = 63) Control group [CG] = 0.5 L/day of semi skimmed milk enriched with vitamins A and D, n = 54	12 months	- EPA and DHA improved bone health by reducing i-PTH levels, which led to decreased RANKL and hs-CRP, lowering bone resorption and reducing inflammation. There were no changes in bone-turnover markers or serum OPG in the intervention group; however, a higher omega-3 to omega-6 ratio was associated with protection against bone mass loss.	Positive
Rajaram, S et al., 2017 [96]	24 healthy adults (15 females and 9 males)	20–70 years Mean age = 42 ± 3 years	- Control diet = 10:1, without supplement - EPA/DHA diet = 10:1, 1.40/5.04g from microalgae oil/week - ALA diet = 2:1, - 42–49 g flaxseed oil/week + 10 g walnuts, three times/week - Combination diet = ALA + EPA/DHA	8 weeks	- Adjusting the n-6:n-3 PUFA ratio or adding EPA/DHA supplements did not impact short-term bone turnover in healthy adults, with no differences observed in bone formation, resorption markers, or PPAR-γ gene expression.	Neutral
LeBoff, MS et al., 2020 [95]	771 (7.9% had fracture history, 80 had osteoporosis, 402 had osteopenia)	Men ≥ 50 years and women ≥ 55 years Mean age 63.8 ± 6.1	Vitamin D + ω-3 PUFAs 1 g/d)	2 years	- No benefit was observed in participants with osteoporosis, as there were no changes in bone-strength indices (polar stress strength index, bone-strength index) or bone structure (total, cortical, and trabecular vBMD, cortical thickness) between the supplement and placebo groups. Furthermore, ω-3 fatty acid supplementation had no effect on aBMD at the spine, femoral neck, total hip, or whole body.	Neutral
Papandreou, P et al., 2020 [106]	66 very low-birth-weight (VLBW), preterm neonates	Gestational age < 32 weeks admitted within 12 h after birth	Soybean oil–based parenteral lipid emulsions [PLE] = Intralipid containing soybean oil (20 g%), egg yolk phospholipids (1.2 g%), glycerin (2.25 g%), and α-tocopherol (38 mg/L) (n = 35) n-3/MCT-enriched PLE = Smoflipid containing fish oil (3 g%), soybean oil (6 g%), olive oil (5 g%), MCTs (6 g%), egg yolk phospholipids (1.2 g%), glycerin (2.5 g%), and α-tocopherol (200 mg/L)	20 days	- ω-3 PUFAs enriched PLEs reduced early bone loss in VLBW neonates by maintaining higher OPG/sRANKL levels during the early postnatal period.	Positive
Ichinose, T et al., 2021 [107]	87 healthy Japanese elderly people	69.1 ± 5.3 years	Placebo group = 200 mL of milk (n = 41) DHA group = 200 mL of milk beverage containing 0.297 g DHA and 0.137g EPA (n = 46)	12 months	- Even low-dose, long-term daily intake of DHA benefited bone health by reducing serum bone-resorption markers. Bone resorption decreased, as indicated by a significant reduction in TRACP-5b.	Positive
Jorgensen et al., 2021 [92]	132 Adult kidney transplant recipients	>75 years	Intervention group = 2.6 ω-3 PUFAs supplements (0.460 g EPA + 0.380 g DHA) Placebo = olive oil	44 weeks	- No changes were observed in BMD at the whole body, lumbar spine, proximal femur, or forearm between the intervention and control groups. Additionally, there were no changes in plasma levels of marine ω-3 PUFAs, TBS, or biochemical parameters of mineral metabolism.	Neutral
Razny, U et al., 2021 [101]	64 Middle-aged individuals with abdominal obesity	25−65 years	Placebo (corn oil) 0.004g of vitamin E per capsule ω-3 PUFAs capsules = 1.8 g DHA + EPA in a ratio of 5:1 Low-calorie diet = 1200 kcal/day for women and 1500 kcal/day for men + ω-3 PUFAs capsules	3 months	- ω-3 PUFAs supplementation had no effect on bone-resorption marker CTX-I, vitamin D levels, osteopontin, FGF-21, or bone-formation markers (PINP, Gla-OC, Glu-OC). Even with calorie restriction, ω-3 PUFAs did not mitigate bone resorption, suggesting limited impact on bone turnover.	Neutral
Matsuzaki, K et al., 2023 [116]	52 Healthy Japanese adults	Mean age = 54.2 ± 6.4 years	Placebo group = 7.0 mL of olive oil daily (n = 25) Intervention group = Perilla frutescens seed oil (PO) = 7.0 mL daily (n = 27)	12 months	- Long-term PO intake improved age-related BMD decline by reducing bone resorption, as evidenced by decreased TRACP-5b levels in the PO group. Additionally, %YAM levels increased compared to placebo, indicating improved bone density. However, there was no change in bone-formation biomarker BALP levels in the PO group.	Positive
S. Gaengler et al., 2024 [117]	1493 older adults (12% osteoporosis, 55% osteopenia, 30% healthy bone density)	≥70 years Mean age = 75 years	1 g EPA+DHA (1:2 ratio, 2 capsules/day)	3 years	- EPA and DHA supplementation showed no significant effect on lumbar spine BMD, femoral neck BMD, or total hip BMD in healthy, vitamin D-replete, active adults. However, when combined with a simple home-based exercise program (SHEP), omega-3 supplementation slightly improved lumbar spine TBS.	Neutral

Abbreviations: AA: arachidonic acid, ALA: Alpha-linolenic acid, BALP/BAP: bone alkaline phosphatase, BMD: Bone mineral density, CTX-I: C-terminal telopeptide of type I collagen, DHA: docosahexaenoic acid, DPD: Deoxypyridinoline, EPA: eicosapentaenoic acid, FGF-21: fibroblast growth factor 21, Gla-OC: carboxylated osteocalcin, IL-6: interleukin-6, LA: linoleic acid, MCT: medium-chain triglyceride, OPG osteoprotegerin, PGE2: prostaglandin E2, PINP: procollagen I N-terminal propeptide, PTH: parathyroid hormone, RANKL: receptor activator of nuclear factor-κB ligand, SCTX: Serum C-Terminal Telopeptide, TBS: trabecular bone score, TNFα: tumour necrosis factor α, TRACP-5b: tartrate-resistant acid phosphatase-5b, YAM: Young Adult Mean.

**Table 3:** Summary of outcomes of Epidemiological studies published between 2010 and 2024 on bone health utilizing diets rich in ω-3 FAs.

Reference	Sample Size (n) and Population	Age/Mean ± SD Age	Treatment (Consisting of ω-3 FA)	Duration	Results	Overall Outcome
Chen, YM et al., 2010 [125]	685 postmenopausal Chinese women.	48–63 years	Mean sea fish intake in quantile categories Q1 = 0.6 g/day (n = 129) Q2–Q4 = 16.8 g/day (n = 420) Q5 = 64.7 g/day (n = 136)	12 years	- Higher sea fish consumption significantly increased mean BMDs. Women in the highest quantile (Q5) of sea fish intake had 4.0% greater whole-body BMD, 4.1% higher lumbar spine BMD, and 6.2% to 8.4% higher BMD at hip sites compared to those in the lowest quantile (Q1).	Positive
Farina, EK et al., 2011 [133]	623 women	Mean age = 78.1 ± 6.88 y	Quartiles of fatty acid intakes for categorical analysesQ1, Q2, Q3, Q4 Low: <one serving/wk Moderate: one to three servings/wk High: ≥three servings/wk	4 years	- Women with elevated EPA+DHA intakes showed increased FN-BMD in the highest intake group (Q4) compared to the lowest (Q1). Conversely, men with lower EPA+DHA intake had decreased FN-BMD.	Positive
Farina, EK et al., 2011 [134]	904 (552 women and 352 men)	Mean age = 75 years	Quartiles of fatty acid intakes for categorical analyses - Low (<1 serving/wk) - Moderate (At least one serving per week but less than three servings per week) - High (≥three servings/wk) one serving of fish equivalent to 85–142 g (3–5 oz).	17 years (1988–2005)	- No effect in hip fracture risk was observed with EPA, DHA, EPA+DHA, or fish intake.	Neutral
Järvinen, R et al., 2012 [135]	554 postmenopausal women	Mean age = 68 years	EPA and DHA = 0.41 ± 0.47 g/day PUFA = 8.8 ± 3.4 g/day MUFA = 17.0 ± 6.3 g/day	3 years	- PUFA intake increased BMD at the lumbar spine and total body, but not at the femoral neck. Higher total PUFA intake increased vitamin D levels, leading to higher lumbar spine and total body BMD. This association is observed in non-Hormone Therapy (HT) users, while no significant link is found for current HT users.	Positive
Harris, TB et al., 2015 [24]	1438 older men and women (540-with fracture, 898 without fracture)	66–96 years	- Never consumed fish oil (referent group) - Less than daily (one time/mo, one to three times/mo, one to two times/mo, or five to six times/wk) - Daily consumption of fish oil	Median follow-up 7.0 year (4.1–7.6 year)	- High ω-3 reduced fracture risk by 34% in men, while daily fish oil intake in midlife reduced fracture risk in women. - High EPA levels reduced fracture risk by 41% in men.	Positive
Choi, E and Park, Y et al., 2016 [119]	- 7154 KNHANES:Korean participants - 2658, NHANES: American participants	≥50 years	Quantiles of Consumption of Fish and Shellfish Q1–Q5	KNHANES-2008 to 2011 NHANES -2007 to 2010	Fish consumption increased BMD in the total femur, femoral neck, and lumbar spine in Korean men and postmenopausal women, while no effects on BMD were observed in American men or postmenopausal women.	Positive
Kuroda, T et al., 2017 [131]	275 healthy Japanese females with peak bone mass (PBM)	19–25 years Mean age = 20.6 ± 1.4 years	- n-3 fatty acid intake 1.3 g/day - Median value of n-3 fatty acid intake = 2.12 g/day (EPA = 0.15 g/day, DHA = 0.25 g/day).	NA	- ω-3 fatty acids increased peak bone mass (PBM) at the total hip BMD. Body mass index (BMI) and serum bone alkaline phosphatase levels were significant contributors to lumbar BMD according to multiple regression analysis.	Positive
Lavado-García et al., 2018 [129]	Total 1865 - women with osteoporosis (n = 194) - with osteopenia (n = 707) - healthy females- (n = 964)	20–79 years	Omega-3 acids - - Linoleic acid = 1.79 g/day - EPA = 0.22 g/day - DHA = 0.30 g/day - ALA = 0.79 ± 0.5 g/day	NA	- DHA intake increased lumbar spine and hip BMD in healthy women, whereas no differences in lumbar spine and hip BMD was observed with ω-3 PUFAs in osteoporotic women.	Positive
Fang, ZB et al., 2023 [4]	8942	20–59 years	Fatty acid intake divided by quartile- Q1–Q4 - Total SFAs intake = 0.6000–190.0570 g/d - Total MUFAsintake = 0.6745–149.8885 g/d - Total PUFAs intake = 0.2825–143.5885 g/d	NA	- PUFAs intake is positively linked to BMD in the third and fourth quartiles, which represent the highest intake levels.	Positive
Feehan, O et al., 2023 [132]	300 Postmenopausal women	45–75 years Mean age 65 years	High n−6: n−3 ratio = ≥6.5 Medium n−6: n−3 ratio = between 4.9 and 6.5 Low n−6: n−3 ratio = ≤4.9	NA	- ω-3 PUFAs, EPA, DPA, and DHA have no effect on bone turnover markers, femur and lumbar spine BMD, or T-score, including total ω-3 PUFAs.	Neutral
Del Brutto, OH et al., 2024 [127]	399 older adults 37% (n = 149) with osteopenia 39% (n = 56) with osteoporosis 24% (n = 94) with normal BMD	≥60 years Mean age 68.8 ± 6.8 years	- High consumption ≥ 5.2 servings/week [728 g] - Low consumption ≤ 5.2 servings/week - Mean dietary intake of oily fish = 8.8 ± 4.8 servings per week	NA	- High fish consumption (≥ 5.2 servings/week) decreased the risk of lower BMD by over two times. - Consuming ≥ five servings/week of wild-caught oily fish reduced the frequency of osteopenia and osteoporosis in older women. - In women, a significant association was observed between high oily fish intake and BMD, not in men.	Positive
Liu, Y et al., 2024 [130]	22,834 13% (n = 2831) patients with OA 87% (n = 20,003) with non-OA	≥20 years	ω-3 PUFAs included: DHA, DPA, EPA, SDA, and ALAOmega-3 Intake Quartiles for Total Omega-3 (g/day): Q1-Reference Q2-0.94 g/day Q3-0.82 g/day Q4-0.79 g/day	NA	- Higher intake of ω-3 PUFAs is associated with reduced OA prevalence, particularly in adults aged 40–59.	Positive

Abbreviations: AA: arachidonic acid, ALA: Alpha-linolenic acid, BMC: bone mineral content, BMD: Bone mineral density, CTX: C-terminal telopeptide, DHA: docosahexaenoic acid, DPA: Docosapentaenoic Acid, EPA: eicosapentaenoic acid, FN-BMD: femoral neck-bone mineral density, IVW: inverse variance-weighted, KNHANES: Korean National Health and Nutrition Examination Survey, LA: linoleic acid, NHANES: National Health and Nutrition Examination Survey, OA: osteoarthritis, OC: osteocalcin, SDA: Stearidonic acid, SFA: saturated fatty acids.

**Table 4:** Summary of outcomes of preclinical studies published between 2016 and 2024 on age-related neurological conditions utilizing diets rich in ω-3 FAs.

Reference	Sample Size (n) and Population	Treatment	Duration	Results	Overall Outcome
Wu, K et al., 2016 [150]	C57BL/6 fat-1/APP transgenic mice	Feeding a high n-6 PUFAs diet to transgenic mice with the fat-1 gene, which converts n-6 PUFAs to ω-3 PUFAs.	12 months	- High PUFA ratios and endogenous ω-3 PUFAs improved AD symptoms by reducing sensorimotor dysfunction and cognitive deficits in mouse models. - n-3 PUFAs decreased amyloid beta aggregation, inflammatory activation, nuclear factor-kappa B activation and neuronal death in APP/fat-1 mice, which also showed lower anxiety levels in behavioral tests.	Positive
Chen, J et al., 2017 [156]	8-week-old male mice	Groups FO1 = 400, 200, and 100 mg of fish oil per kg of body weight per day Group DHA = 120, 60, and 30 mg of DHA per kg of body weight per day	2 months	- ω-3 PUFAs enhanced aging resilience by improving redox homeostasis, increasing antioxidant enzymes (superoxide dismutase and catalase), and lowering oxidative stress markers (monoamine oxidase and F2-isoprostanes). - ω-3 PUFAs protected against DNA damage and telomere shortening, with DHA inhibiting cellular senescence, promoting healthier aging.	Positive
Prokopiou, E et al., 2019 [154]	24-month-old aged wild-type C57BL/6J mice	three groups (n = 15/group): - Young untreated - Aged untreated - Aged treated with ω-3 PUFAs (571 mg/mL EPA and 114 mg/mL DHA fish formulation)	2 months	- ω-3 supplementation reduced age-related retinal degeneration in mice by decreasing lipofuscin granule formation and protecting the photoreceptor layer, effectively slowing normal aging processes.	Positive
Varela-Lopez et al., 2022 [155]	72 male Wistar rats weighing 80–90 g	n-3 fatty acid profile of experimental dietary fats - olive oil = 0.4 g/100 g - Sunflower oil = 0.4 g/100 g - Fish oil = 31.3 g/100 g	24 months	- Fish oil did not show a major effect on preserving the liver during aging. It intensified age-related oxidation, reduced electron transport chain activity, and enhanced relative telomere length.	Neutral
Xia, J et al., 2023 [152]	3-month-old adult male and 24-month-old male aging Sprague–Dawley (SD) rats	3 groups: - Aging (saline) - Aging + EPA = 500 mg/kg/day EPA - Aging + DHA = 500 mg/kg/day DHA	8 weeks	- DHA outperformed EPA in mitigating age-related neuroimmunological changes and enhancing memory. ω-3 PUFAs, especially DHA, corrected microglial M1/M2 polarization in the hippocampus, improving spatial memory in aging rats. DHA significantly lowered TNF-α, IL-1β, and IL-6 levels, while EPA primarily suppressed IL-6, emphasizing DHA’s neuroprotective benefits.	Positive
Wang, X et al., 2024 [151]	Male Senescence Accelerated Mouse-Prone 8 (SAMP8) mice (3–5-month-old	endogenous and exogenous DHA milk powder containing 0.33% DHA.	42 days	- 0.33% DHA reduced oxidative stress in the brains and serum of SAMP8 mice, evidenced by increased SOD activity. Endogenous DHA milk outperformed exogenous DHA milk in improving liver SOD activity, oxidative stress levels, and cognitive abilities, as shown by improved performance in the water maze test.	Positive

Abbreviations: AA: arachidonic acid, APP: amyloid precursor protein, DHA: Docosahexaenoic acid, EPA: Eicosapentaenoic acid, GSH-Px: glutathione peroxidase, SOD: superoxide dismutase.

**Table 5:** Summary of outcomes of clinical studies published between 2010 and 2024 on age-related neurological conditions utilizing diets rich in ω-3 FAs.

Reference	Sample Size (n) and Population	Age/Mean ± SD Age	Treatment	Duration	Results	Overall Outcome
Yurko-Mauro, K et al., 2010 [158]	485 healthy Adults with ARCD	≥55 years	0.9 g/d of DHA	24 weeks	- 0.9 g/d DHA enhanced learning and memory function, reduced errors in the PAL test, and improved immediate and delayed VRM scores, leading to a twofold decrease in visuospatial learning and episodic memory errors.	Positive
Sinn et al., 2012 [157]	50 Patients with MCI	>65 years	- Control (safflower oil) = 2.2 g LA/d - EPA-rich fish oil = 1.67 g EPA + 0.16 g DHA/d - DHA-rich fish oil = 1.55 g DHA + 0.40 g EPA/d	6 months	- DHA and EPA improved mental health in older adults with MCI, enhancing verbal fluency and self-reported physical health. - DHA and EPA intake improved GDS scores, but no effects were observed on other cognitive or quality-of-life measures.	Positive
Eriksdotter, M et al., 2015 [166]	174 AD patients	74 ± 9 years	2.3 g ω-3 PUFAs	6 months	- Higher plasma levels of ω-3 FAs reduced cognitive deterioration in AD patients.	Positive
Taghizadeh, M et al., 2017 [163]	60 patients with PD	50–80 years	1 g ω-3 PUFAs from flaxseed oil plus 400 IU vitamin E supplements	3 months	- ω-3 FAs and vitamin E co-supplementation improved UPDRS scores in PD patients but had no effect on inflammation, oxidative stress biomarkers, or lipid profiles, and resulted in decreased insulin levels.	Positive
Tamtaji, OR et al., 2019 [162]	40 adults with PD	50–80 years	1 g daily of ω-3 PUFAs from flaxseed oil + 400 IU/day of vitamin E	12 weeks	- ω-3 FAs and vitamin E co-supplementation improved the gene expression of TNF-α, PPAR-γ, and LDLR, indicating beneficial anti-inflammatory and metabolic effects that positively manage PD. - Significantly improved UPDRS-part I.	Positive
Arellanes, IC et al., 2020 [160]	33 with a first-degree family history of dementia	≥55 years	- Placebo (n = 15, APOE4 = 7, non-APOE4 = 8) - Intervention group IG = 2.152 g/day of TG-based DHA supplementation (n = 18, APOE4 = 8,non-APOE4 = 10)	6 months	- Doses of 1 g/day or less of ω-3 FAs showed limited effects on brain health in dementia prevention trials. DHA treatment increased CSF DHA levels by 28% and EPA levels by 43%.	Positive
Pantzaris, M et al., 2021 [164]	40 mild to moderate severity PD patients	40–75 years old	- Placebo = pure virgin olive oil 20 mL - Neuroaspis PLP10^®^ = a mixture of omega-3 [0.810 g EPA and 4.14 g DHA) and omega-6 fatty acids (1.8 g GLA and 3.15 g LA) (1:1 *w*/*w*)	30 months	- Neuroaspis PLP10™ supplementation in PD patients significantly delayed disease progression, as evidenced by an increase in UPDRS III scores. These positive effects persisted throughout the study, lasting until the end of the 30-month period.	Positive
Power, R et al., 2021 [159]	60 cognitively healthy adults	≥65 years	- Control group, Placebo = sunflower oil - Intervention group (IG) = 1 g fish oil (430 mg DHA, 90 mg EPA), 22 mg carotenoids (10 mg lutein, 10 mg mesozeaxanthin, 2 mg zeaxanthin, and 15 mg vitamin E)	24 months	- DHA and EPA improved working memory, resulting in significantly fewer errors in tasks compared to placebo. - DHA and EPA showed improvements in attention, language, and global cognition following the intervention.	Positive
Del Brutto, OH et al., 2022 [165]	263 individuals of Amerindian ancestry	≥60 years	Mean oily fish intake was 8.3 ± 4 servings per week	6.5 years	- High fish oil intake benefitted brain health and reduced neurodegeneration by providing a protective effect against the progression of WMH.	Positive
Lin, PY et al., 2022 [33]	163 MCI or AD patients	77.8 (treatment) vs. 78.1 (placebo)	Placebo = soybean oil (n = 40) DHA = 0.7 g/day (n = 41) EPA = 1.6 g/day, (n = 40) EPA (0.8 g/day) + DHA (0.35 g/day) (n = 42)	24 months	- ω-3 FAs supplements did not reduce cognitive, functional, or depressive symptoms, but they did improve spoken language. - EPA intervention improved spoken language ability and constructional praxis, reducing TNF-α, IFN-γ, and IL-1β levels.	Positive
Torres-Mendoza, BMG et al., 2022 [161]	87 AD patients	NA	Fish oil = 0.45 g of EPA and 1 g of DHA	12 months	- ω-3 FAs intake reduced oxidative stress markers (protein, lipid oxidation) in AD patients. ω-3 FAs enhanced the antioxidant defense system in AD patients, as evidenced by the significant increase in catalase activity after 6 and 12 months of fish oil treatment.	Positive

Abbreviations: AD: Alzheimer’s disease, ADAS-Cog: Alzheimer’s Disease Assessment Scale-Cognitive Subscale, ARCD: age-related cognitive decline, BDNF: brain-derived neurotrophic factor, CSF: cerebrospinal fluid, DHA: Docosahexaenoic acid, EPA: Eicosapentaenoic acid, FPG: fasting plasma glucose, GLA: gamma-Linolenic acid, GSH: glutathione, hs-CRP: high-sensitivity C-reactive protein, IFN: interferon, IL: interleukin, LA: Linoleic acid, LDLR: low-density lipoprotein receptor, MCI: mild cognitive impairment, MMSE: Mini-Mental State Examination, PAL: Paired Associate Learning, PD: Parkinson’s disease, TAC: total antioxidant, TNF-α: tumour necrosis factor-α, UPDRS: Unified Parkinson’s Disease Rating Scale, VRM: Verbal Recognition Memory, WMH: white matter hyperintensity.

**Table 6:** Summary of outcomes of Epidemiological studies published between 2013 and 2024 on age-related neurological conditions utilizing diets rich in ω-3 FAs.

Reference	Sample Size (n) and Population	Age/Mean ± SD Age	Treatment	Duration	Results	Overall Outcome
Ammann, EM et al., 2013 [168]	2157 dementia-free women with normal cognition	65–80 years	RBC DHA + EPA content (%): High (n = 719) = 6.44% Middle (n = 719) = 4.90% Low (n = 719) = 3.83%	7.9 years	- DHA + EPA had no effect on age-related cognitive decline in older, dementia-free women, though those in the highest DHA + EPA tertile exhibited improved verbal knowledge, fine motor speed, and verbal fluency.	Neutral
Gustafson, DR et al., 2020 [171]	2612 adults without dementia	76 years	Tertiles of fatty acid intake T1, T2, T3 - Long-chain saturated fatty acids - Polyunsaturated fatty acids (DHA EPA) - Monounsaturated fatty acids	4.9 years	- DHA and EPA reduced Alzheimer’s disease risk by 27% and 26%, respectively. - Participants experienced longer periods without AD (*p* < 0.05).	Positive
Nozaki, Shoko et al., 2021 [169]	1127 Japanese individuals 468 men, 659 women	73 years	- Quartiles of fish consumption ω-3 PUFAs, EPA, DHA, EPA+DHA, DPA, ALA, n-6 PUFAs, LA, ARA	15 year	- Higher quartiles of fish, EPA, DHA, and DPA intake showed significantly reduced dementia risks.	Positive
Wei, BZ et al., 2023 [172]	ADNI study n = 1135 - 828 Participants free of AD - 307 Participants who developed AD,	55–90 year Mean age = 73.36 ± 7.22 y)	Omega-3 supplementation - Exposure (yes or no) Medium term exposure (1~9 year) - long-term exposure (10 year)	6 years mean follow-up time of 2.81 ± 1.60 year	- Long-term use of ω-3 PUFA supplements reduced the risk of Alzheimer’s disease by 64%.	Positive
Li, Benchao et al., 2024 [167]	2621 older Chinese adults with MCI	≥45 years	Aquatic food consumption Quartile 1: 0–12.38 g/d Quartile 2: 12.39–28.33 g/d Quartile 3: 28.34–56.20 g/d Quartile 4: 56.20 g/d	4.5–6.3 years	- Consumption of aquatic foods and higher intake of ω-3 PUFA reduced the risk of mild cognitive impairment (MCI) and improved cognitive function.	Positive

Abbreviation: ADRD: Alzheimer’s Disease and Related Dementias, DHA: Docosahexaenoic acid, EPA: Eicosapentaenoic acid, MCI: mild cognitive impairment.

## Data Availability

Not applicable.
